# Local conditions drive interpopulation variation in field-based critical thermal maximum of brook trout

**DOI:** 10.1093/conphys/coae086

**Published:** 2024-12-26

**Authors:** Erin M C Stewart, Jacob C Bowman, Chris C Wilson, Graham D Raby

**Affiliations:** Environmental and Life Sciences Graduate Program, Trent University, 1600 West Bank Drive, Peterborough, Ontario K9L 0G2, Canada; Department of Biology, Trent University, 1600 West Bank Drive, Peterborough, Ontario K9L 0G2, Canada; Ontario Ministry of Natural Resources, Aquatic Research and Monitoring Section, Trent University, 2140 East Bank Drive, Peterborough, Ontario K9L 0G2, Canada; Environmental and Life Sciences Graduate Program, Trent University, 1600 West Bank Drive, Peterborough, Ontario K9L 0G2, Canada; Department of Biology, Trent University, 1600 West Bank Drive, Peterborough, Ontario K9L 0G2, Canada

**Keywords:** Acclimation, Body size, Climate change, Field CT_max_, Intraspecific variation, Local adaptation, Plasticity, Salmonid*, Salvelinus fontinalis*, Thermal tolerance

## Abstract

Individual- and population-level responses to thermal change will be pivotal for species’ resilience and adaptive responses to climate change. Thermal tolerance of ectotherms has been extensively studied under laboratory conditions, but comparatively few studies have assessed intra- and interpopulation variation under natural conditions or *in situ*. We measured field critical thermal maximum (CT_max_) of brook trout (*Salvelinus fontinalis*) populations at twenty sites across Ontario, Canada, to assess their thermal tolerance *in situ* and examine potential factors underlying intraspecific variation in thermal performance. We modelled CT_max_ as a function of acclimation using short-term stream temperature data to assess interpopulation variation, and used full-season stream temperatures to calculate thermal safety margins (TSM) for each population. CT_max_ ranged between 27.41 and 30.46°C and acclimation periods between 4 and 40 days were strong predictors of site CT_max_, aligning closely with lab-based studies. Seasonal temperature profiles varied substantially among sites, with mean 30-day stream temperature accounting for 66% of the among-site variation in CT_max_. TSMs ranged between 0.51 and 15.51°C and reflected differences among site thermal regimes. Streams in watersheds with more urban or agricultural development had the lowest TSMs in addition to those that were fed by lake surface water. This work emphasizes the importance of locally based conservation and management practices that act at or below the population level, as local factors beyond acclimation temperature were partly responsible for variation in thermal tolerance and thus dictate the resiliency of brook trout under climate change.

## Introduction

Species-level responses to shifting environmental conditions are driven by changes within populations. Management practices that conserve genetic and phenotypic diversity are therefore important for maximizing their resilience. The ecology of many fish species is heavily influenced by temperature (Brett, 1971), so phenotypic diversity in thermal performance is key for resilience and the potential for adaptation under climate change (Pörtner and Farrell, 2008; Seebacher *et al.*, 2015; Norin *et al.*, 2016). Indeed, some fishes exhibit remarkable intraspecific variation in thermal tolerance at the individual, family and population levels (Fields *et al.*, 1987; Eliason *et al.*, 2011; McDermid *et al.*, 2012; Stitt *et al.*, 2014; Chen *et al.*, 2018). The three primary mechanisms driving intraspecific variation in thermal tolerance are heritable genetic differences, plasticity, and ontogenic shifts through body sizes and life stages (McKenzie *et al.*, 2021). For many species, however, information is lacking about how much intraspecific variation in thermal tolerance occurs naturally. Although some studies have assessed intraspecific variation in thermal performance under controlled conditions (Fields *et al.*, 1987; Eliason *et al.*, 2011; McDermid *et al.*, 2012; Stitt *et al.*, 2014; Chen *et al.*, 2018), *in situ* assessments of upper thermal tolerance across populations can allow us to quantify variation under natural conditions and investigate its potential causes.

Critical thermal maximum (CT_max_) is a useful metric that provides a relative measure of thermal resiliency and a margin for thermal safety in the face of climate change. Most CT_max_ assays have been done under laboratory conditions, but a small number have been done *in situ* (Gilbert *et al.*, 2020; Leclair *et al.*, 2020; Turko *et al.*, 2020; Firth *et al.*, 2021; Reemeyer *et al.*, 2024). Conducting CT_max_  *in situ* is advantageous because it can capture natural conditions that influence thermal tolerance, such as variable acclimation temperatures due to diel fluctuations or irregular pulses, as well as changing food availability, the presence of predators, and other environmental covariates (Leclair *et al.*, 2020). Doing so can also generate a population-specific thermal safety margin (TSM), which is the difference between CT_max_ and the highest ambient temperature the population faces in nature during a given period, typically within a given season, but definitions of the highest temperature vary across studies (e.g., mean temperature of warmest month in Comte and Olden, 2017; field acclimatization temperature at time of CT_max_ trial in Firth *et al.,* 2021; daily maximum temperature on date of CT_max_ trial in Leclair *et al.,* 2020; overall acclimation temperature in Sandblom *et al.,* 2016 and McArley *et al.,* 2017).

Of the many challenges facing coldwater species, increased environmental temperatures due to climate change and land use change is the most widespread (Hudy *et al.*, 2008; Stranko *et al.*, 2008; Siitari *et al.*, 2011; Argent and Kimmel, 2013). Brook trout (*Salvelinus fontinalis*), one of North America’s most iconic coldwater species, occurs in variable thermal environments across their range, occupying a diverse set of lotic and lentic habitats that range from small groundwater-fed streams to the Laurentian Great Lakes (Hutchings, 1993; Quinn *et al.*, 1994; Borwick *et al.*, 2006; Ridgway, 2008; Petty *et al.*, 2012). Where watershed connectivity is naturally low or has been reduced by thermal barriers or dams, brook trout populations are genetically distinct and may be adapted to local conditions (Letcher *et al.*, 2007; Whiteley *et al.*, 2013; Kelson *et al.*, 2015). While there is some evidence of local adaptation in brook trout, intraspecific variation in life history and thermal tolerance can also be driven by plasticity (Baird *et al.*, 2002; Morinville and Rasmussen, 2003; Letcher *et al.*, 2007; Ridgway, 2008; McDermid *et al.*, 2012; Stitt *et al.*, 2014; Cook *et al.*, 2018).

Thermal tolerance in brook trout has been studied extensively *ex situ,* predominantly on juvenile fish (often ages 0–1+) and on hatchery-reared populations (reviewed in Smith and Ridgway, 2019). For a coldwater fish, brook trout have a notably wide and flat aerobic scope, where aerobic performance is optimized around 15°C, but is relatively even across temperatures from 5 to 20°C (Graham, 1949; Basu, 1959; Beamish, 1964; Stitt *et al.*, 2014; Durhack *et al.*, 2021). Upper thermal tolerance in brook trout is plastic with thermal acclimation; CT_max_ has been reported between *ca.* 26–31°C when acclimated between 5 and 25°C (Lee and Rinne, 1980; Benfey *et al.*, 1997; Galbreath *et al.*, 2004; McDermid *et al.*, 2012; Stitt *et al.*, 2014; Wells *et al.*, 2016; Morrison *et al.*, 2020; O’Donnell *et al.*, 2020; Mackey *et al.*, 2021; Lechner *et al.*, 2024). CT_max_ is a repeatable trait in brook trout (O’Donnell *et al.*, 2020) and continues to increase with thermal acclimation within the optimal range for at least 30 days (Stewart *et al.*, 2023). Evidence of intraspecific variation in brook trout thermal tolerance exists at the family (Stewart *et al.*, 2023) and population levels (McDermid *et al.*, 2012; Stitt *et al.*, 2014; Stewart *et al.*, 2024) under controlled conditions, indicating a heritable basis to their thermal performance as well as the potential for adaptive responses within and among populations based on existing variation. Given how diverse brook trout are, both physiologically and geographically (Hutchings, 1993; Quinn *et al.*, 1994; Fraser and Bernatchez, 2008; Ridgway, 2008), local adaptation is likely to be an important mechanism causing intraspecific variation in the species (Harbicht *et al.*, 2014; Zastavniouk *et al.*, 2017). To date, however, there has been no systematic effort to assess among-population differences in upper thermal tolerance in brook trout *in situ* or at a broad geographic scale.

The objective of this study was to generate a robust assessment of brook trout thermal tolerance that examines the drivers of intraspecific variation in thermal tolerance *in situ*. We conducted CT_max_ trials on twenty populations of brook trout across Ontario and paired those data with short- and long-term stream temperature data to assess plasticity and local adaptation and generate population-specific thermal safety margins. We hypothesized that differences in upper thermal tolerance among populations arise as a result of thermal acclimation (plasticity) and local (genetic) adaptation. We predicted that CT_max_ would increase with increasing acclimation temperatures and that residual variation would occur, driven by population-level differences after controlling for acclimation (plasticity). We also predicted that TSMs would vary widely across the sites, with the influence of groundwater and urban development as likely causes for differences driven by stream thermal regimes. The data presented here ultimately help illustrate variation in thermal tolerance among populations of an iconic coldwater fish. Our hope is that the population-specific estimates will be useful for conservation planning while furthering our understanding of the drivers of intraspecific variation in thermal tolerance of ectotherms.

## Materials and Methods

**Figure 1:**
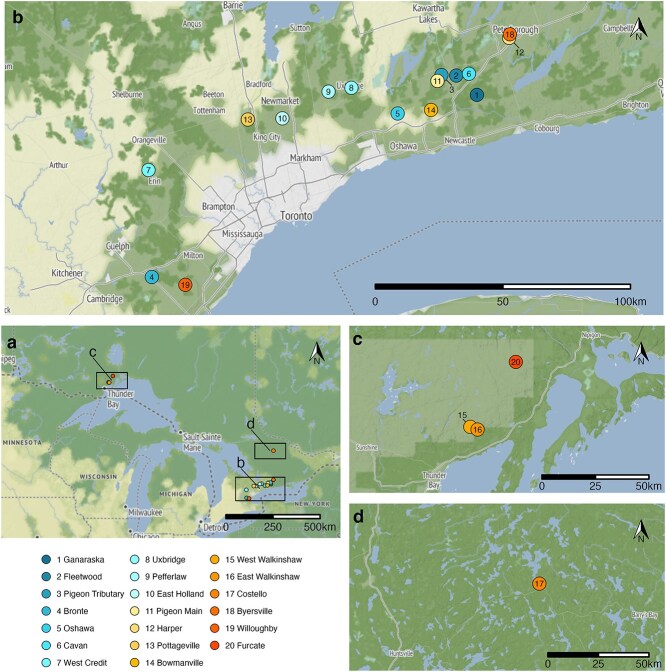
Twenty sites in Ontario, Canada where *in situ* CT_max_ was conducted on wild brook trout (**a**). Sites were concentrated in three regions: south (**b**), north (**c**) and Algonquin (**d**). Most sites were in the southern region of the province (b; 16 sites). Three sites were in the northern region (c), and one in the Algonquin region (d). Sites are numbered and coloured based on 30-day mean stream temperatures prior to sampling (1 being the lowest, 20 being the highest). Basemap colouration is a broad index of land cover, where white depicts a high degree of urban and residential development, light yellow is predominantly agricultural and light to dark green depicts increasing tree cover over less developed areas (e.g., with small-scale agriculture, forestry, protected areas). Maps created using *ggmap* package and basemaps from Stadia Maps and Stamen Maps (Kahle and Wickham, 2013; stadiamaps.com; stamen.com).

This study was conducted between June and October of 2021 at 20 sites across Ontario (Fig. 1). All fish collection and handling was approved by the Trent University Animal Care Committee following guidance set by the Canadian Council on Animal Care (Trent U AUP #26398) and collection permits issued by the Ontario Ministry of Natural Resources (OMNR) to G.D. Raby and partner agencies involved with fish collections (OMNR and Ontario Parks Licence to Collect Fish for Scientific Purposes to G.D. Raby #1098026). Ten of the 20 sites were studied in collaboration with partner agencies who conduct long-term monitoring and research at the sites (Supplementary S1).

Our objective with site selection was to capture a broad range of genetic, geographic, and thermal variation. We considered watershed characteristics that would relate to each site’s thermal regime, such as relative forest cover, proximity to urban areas, historic watershed connectivity, and watershed source (groundwater or lake surface water), as well as genetic sources of variation or selection based on historic or contemporary stocking of conspecifics and competitors (Table 1). Records exist at most sites, but the lack of precise watershed stocking location and genetic analysis leaves contemporary genetic ancestry unclear. Brook trout in stocked streams and lakes exhibit varying degrees of genetic introgression following repeated stocking of hatchery-reared conspecifics, with some populations retaining up to 100% native ancestry, (0–100% introgression based on neutral genetic markers; Reaume, 2006; Alshamlih*,* 2014).

**Table 1:** Brook trout site watershed characteristics, genetic ancestry and stocking history. Upstream drainage area (km^2^), lakes/wetlands area (km^2^), mean annual air temperature (°C), tree cover (%; not including plantation), and the top three land cover types (%) of the upstream catchment area at the collection point were obtained using the Ontario Watershed Information Tool (https://www.ontario.ca/page/ontario-watershed-information-tool-owit ). Genetic ancestry and watershed stocking history information is based on provincial records of stocking history, personal communication with Conservation Authorities and landowners, and previous genetic sampling (Reaume, 2006; C. Wilson, unpublished data). Genetic ancestry should be interpreted with caution given the lack of contemporary genetic sampling and the likelihood of unrecorded stocking and escapement on private land. Stocking year ranges indicate earliest and latest provincial records of stocking (i.e., not continuous records). Provincial records prior to 2003 do not indicate location in watershed; therefore, it is unknown if sampled stream reaches would have mixed with stocked conspecifics.

**Site**	**Drainage area (km** ^**2**^ **)**	**Lakes and wetlands (km** ^**2**^ **)**	**Air temp (°C)**	**Tree cover (%)**	**Land cover (%)**	**Genetic ancestry**	**Watershed stocking history**
Bowmanville	4.059	0.824	6.8	37.899	43.4—Agricultural/rural	Unknown	Stocked 1949–1972
					15.4—Coniferous treed		
					12.9—Swamp		
Bronte	7.295	0.671	7.4	5.66	63.4—Agricultural/rural	Unknown	Stocked 1949–1971
					16.0—Urban development		
					11.5—Swamp		
Byersville	4.889	0.08	6.8	9.099	86.1—Urban development	—	None recorded; known history
					3.7—Coniferous treed		
					3.0—Agricultural/rural		
Cavan	49.801	3.253	6.7	35.759	38.6—Agricultural/rural	Introgressed	1946–1986 and likely continued escapement from privately stocked ponds
					15.3—Mixed treed		
					12.5—Deciduous treed		
Costello^a^	14.974	2.274	4	88.199	44.0—Mixed treed	Unknown	Stocked 1950–2007 (upstream lake and creek)
					29.0—Deciduous treed		
					13.5—Coniferous treed		
East Holland	4.021	0.263	7.2	23.401	36.8—Agricultural/rural	Unknown	Stocked 1949–1972
					32.7—Urban development		
					12.8—Deciduous treed		
East Walkinshaw^a^	38.981	5.908	1.9	85.98	32.1—Deciduous treed	Unknown	Stocked 1954 (upstream lake)
					27.5—Sparse treed		
					21.9—Mixed treed		
Fleetwood	5.869	0.31	6.7	68.522	30.8—Mixed treed	Unknown	Stocked 1947–1987
					26.1—Deciduous treed		
					18.9—Agricultural/rural		

(Continued)

**Table 1:** Continued

**Site**	**Drainage area (km** ^**2**^ **)**	**Lakes and wetlands (km** ^**2**^ **)**	**Air temp (°C)**	**Tree cover (%)**	**Land cover (%)**	**Genetic ancestry**	**Watershed stocking history**
Furcate^a^	54.793	6.611	2.2	79.809	27.1—Mixed treed	Unknown	Stocked 1956–2024 (upstream lake; creek twice in 1961)
					26.7—Sparse treed		
					24.6—Deciduous treed		
Ganaraska	7.93	0.047	6.9	49.328	47.3—Plantations treed	Unknown	Stocked 1946–1986
					25.3—Deciduous treed		
					21.1—Mixed treed		
Harper	1.653	0.242	6.9	9.036	68.8—Urban development	—	None recorded; anecdotal history
					12.5—Swamp		
					8.8—Agricultural/rural		
Oshawa	6.308	0.086	6.9	17.878	73.3—Agricultural/rural	Unknown	Stocked 1950–1961
					10.0—Deciduous treed		
					4.3—Coniferous treed		
Pefferlaw	31.767	1.457	7	22.048	43.5—Agricultural/rural	Unknown	Stocked 1947–1972
					10.4—Urban development		
					10.1—Deciduous treed		
Pigeon Main	28.214	2.894	6.6	35.509	35.7—Agricultural/rural	Unknown	Stocked 1946–1972 (creek), 1948–2012 (lake)
					14.3—Mixed treed		
					11.9—Deciduous treed		
Pigeon Tributary	6.055	0.121	6.7	13.046	76.7—Agricultural/rural	Unknown	Stocked 1946–1972 (creek), 1948–2012 (lake)
					5.0—Deciduous treed		
					4.1—Mixed treed		
Pottageville	6.039	0.809	7.1	61.496	29.1—Mixed treed	—	Unclear records; stocking unlikely
					23.9—Deciduous treed		
					19.4—Agricultural/rural		

(Continued)

**Table 1:** Continued

**Site**	**Drainage area (km** ^**2**^ **)**	**Lakes and wetlands (km** ^**2**^ **)**	**Air temp (°C)**	**Tree cover (%)**	**Land cover (%)**	**Genetic ancestry**	**Watershed stocking history**
Uxbridge	17.598	1.112	7	31.673	39.2—Agricultural/rural	Unknown; suspected introgression	Stocked 2003–2021 (upstream pond)
					15.7—Deciduous treed		
					11.9—Coniferous treed		
West Credit	4.792	1.047	6.4	8.893	64.5—Agricultural/rural	Unknown; suspected native	Stocked (various areas) 1946—Present
					23.4—Swamp		
					4.3—Mixed treed		
West Walkinshaw^a^	24.85	5.532	2.1	89.698	48.6—Deciduous treed	Unknown	Stocked Walkinshaw Lake—1954
					31.7—Mixed treed		
					7.1—Sparse treed		
Willoughby	10.839	0.677	8.3	13.462	52.6—Agricultural/rural	Unknown	Stocked 1972
					9.2—Mine tailings/extraction		
					8.0—Urban development		

a Predominantly lake-fed creek

### Fish capture

Fish were captured via backpack electrofishing using voltage, amperage, and frequency settings that were determined on a site-specific basis based on stream conditions and fish health considerations. The length of stream sampled varied by site, ranging from 100 to 300 m as required to achieve a minimum sample size (10 fish). Each site’s sample size was determined by the number of individuals captured before midday (1100–1300 EDT) when CT_max_ trials were commenced (*n* = 10–36 per site). Across the 20 sites, we quantified CT_max_ in 394 fish. Following initial observations of higher rates of injury and mortality in *ca*. 40–70 mm young-of-year brook trout at the first site (that we believe to be due to additional external factors during collection), we set a minimum fork length of 80 mm (approximate; length imprecisely measured during collection to minimize excess handling) for use in the study to comply with allowable mortality under our animal care and collection permits. Upon capture, fish were transferred to the CT_max_ arena, where they were held in ambient, aerated stream water until electrofishing of the site was complete and enough fish were accumulated for the trial to begin. Fish were given between 13 and 324 min to acclimatize to the CT_max_ arena prior to the trial starting (mean acclimatization time = 55 min; mean difference of within-site acclimatization period = 62 min). Arena acclimatization times represent site-level minima and maxima, as individual fish could not be uniquely identified until being removed from the trial arena. The tank water was monitored during the acclimatization period and was refreshed as needed to ensure it remained as close to the ambient stream temperature as possible. Thirteen minutes is enough time for the core temperature of fish of this size to fully acclimate to the arena temperature (O’Donnell *et al.*, 2024).

### Critical thermal maximum trials

CT_max_ trials were conducted streamside using ambient stream water and were conducted simultaneously on all individuals at a given site using the same arena as in Stewart *et al.* (2023). The arena consisted of two connected insulated tanks (113.6 L cooler filled with 100 L of water and 47.3 L cooler filled with 40 L of water): the smaller of the two acted as a sump, housing six 100–500 W aquarium rod heaters, a large air stone, and three Eheim pumps (2 × 300 L h^−1^, 1 × 600 L h^−1^) that recirculated heated and aerated water between the two tanks (see Supplementary S2 for photos and details). The equipment was powered using a gas-powered generator. Trials began between 1030 and 1345 EDT and as close to ambient stream temperatures as possible, subject to slight changes due to surrounding air and ground temperatures (−0.77–1.89°C from ambient stream temperature upon arrival at each site). We used a warming rate of 4°C h^−1^ instead of the commonly used 18°C h^−1^ (0.3°C min^−1^) for two reasons: (i) given the wide range of body sizes within and among sites, the effect of a body temperature lag may be reduced at slower warming rates (Galbreath *et al.*, 2004; Azumaya and Ishida, 2005; Terblanche *et al.*, 2007; Moyano *et al.*, 2017; Pottier *et al.*, 2022; O’Donnell *et al.*, 2024), and (ii) we were limited in the amount of power available to warm the volume of water in the arena. This warming rate is also more ecologically relevant in that it is closer to warming rates that can occur in nature in small streams, such as during extreme heat events driven by urban runoff (Van Buren *et al.*, 2000; Thompson *et al.*, 2008; Cockerill *et al.*, 2017) or low forest cover (McGurk, 1989; Dai *et al.*, 2022). Actual warming rates ranged between 3.89 and 4.09°C h^−1^ (see Supplementary S3 for warming rates plotted for each trial).

Water temperature in the trial arena and sump tank was monitored and recorded throughout the trial at 15 s intervals using an Onset HOBO MX2304 External Temperature Sensor Data Logger (0.2°C accuracy, 0.01°C precision, 20 s response time in water; www.onsetcomp.com). The endpoint was loss of equilibrium (LOE), which we defined as the moment when an individual could no longer maintain or regain positive (vertical) orientation following a gentle nudge (i.e., loss of the righting reflex). The same observer (E.M.C. Stewart) evaluated loss of equilibrium (LOE) in each trial. Upon LOE, fish were immediately removed from the arena using a hand net and moved to uniquely numbered recovery tanks. We recorded the time (to the s) and temperature at the time of LOE (nearest 0.01°C). Recovery tanks were well aerated and at most 21.5°C (approximate range of recovery temperatures = 11.7–21.5°C).

After all fish at a site had at least 10 min in recovery, fish were lightly anaesthetized (stage 3 anaesthesia) using tricane methanesulfonate (80 mg L^−1^ MS-222; Syndel, Nanimo, B.C., Canada) to reduce handling stress while fork length (mm) and mass (g) were measured, and a scale sample from the subdorsal lateral area was taken for future study. Once recovered from anaesthesia, all fish were released back into the stream. There were 10 mortalities during the recovery period following CT_max_ (i.e., fish were not able to regain equilibrium). These fish were not included in analyses.

### Acclimation periods

We recorded stream temperatures to capture the acclimation regimes for each population. As a result of our collaborations with other agencies for access to the populations, stream temperatures were recorded using varying logger types and logging intervals, and for variable durations (Supplementary S1). Loggers were deployed at the approximate area where fish were expected to be captured at least 14 days in advance of the CT_max_ trial at most sites (16 of 20), and at least 30 days in advance at half of the sites (10 of 20). To account for sites with less than 14 days of acclimation data, and to expand our analysis beyond 14 days, we imputed the missing data using the existing temperature logger data and the relationship between air temperature and water temperatures. We extracted air temperature data from the Environment and Climate Change Canada Historical Climate Data web site (https://climate.weather.gc.ca/index_e.html) on 18 March 2024 for both the time periods corresponding to the existing stream temperature data and the missing data (ECCC, 2024). We used air and water temperatures from May to October because the relationship between the two variables becomes non-linear when air temperature declines below *ca.* 0°C (Erickson and Stefan, 2000). Since the warming effect of air temperature on stream temperature causes peaks in stream temperature to lag behind air temperature in time, we introduced a lag in stream temperature into our models. We extracted hourly data from the nearest weather station to each site and tested the time lag between air temperature and stream temperature that produced the best linear model. Using the best time lag interval, we produced two candidate models of stream temperature for each stream (see Supplementary S4 for regression terms for each site).

First, we performed linear regression analysis, where *T_stream_* is stream temperature (°C), *T_air(lag)_* is the air temperature lagged in time after stream temperature (°C), *m* is the increase in *T_stream_* for every 1°C increase of *T_air(lag)_*, and *b* is the value of *T_stream_* when *T_air(lag)_* is 0°C (Equation 1). Linear models have frequently been used to reliably calculate stream temperature from air temperature (Benyahya *et al.*, 2007; Laanaya *et al.*, 2017). Second, we fit a logistic function to the relationship between air temperature and stream temperature, where *T_stream_* is stream temperature (°C), *T_air(lag)_* is air temperature lagged in time after stream temperature (°C), *L* is the maximum value of the curve, *k* is the logistic growth rate, and *x_0_* is the sigmoidal inflection point (Equation 2). We fit logistic curves to temperature data using the Levenberg–Marquardt Nonlinear Least-Squares Algorithm in R (R Core Team, 2022, version 4.2.0; *minipack.lm* package, Elzhov *et al.*, 2024). Logistic regression is another widely used method for predicting stream temperature from air temperature (Piotrowski and  Napiorkowski, 2019), and incorporates the S-shaped effects of extreme cold and extremely warm air temperatures on stream temperatures (Mohseni *et al.*, 1998).


(1) \begin{equation*} {T}_{stream}=m\left({T}_{air(lag)}\right)+b \end{equation*}

(2) \begin{equation*} {T}_{stream}=\frac{L}{1+{e}^{-k\left({T}_{air(lag)}-{x}_0\right)}} \end{equation*}

After fitting both linear and logistic models, we chose the model that best predicted the observed 2021 stream temperatures by performing linear regressions between observed temperatures and predicted temperatures and picking the model that produced the highest *R*^2^ (Supplementary S4). Using the best model for each stream (18 logistic, 2 linear; mean best-fit *R*^2^ = 0.64), we extrapolated stream temperatures from May to October using air temperature. We used the back-calculated data to complete the hourly stream temperature datasets for all sites, but did not replace recorded data with back-calculated data (12 of 20 sites had over 75% of the period recorded; Supplementary S1). For three sites where temperature logger data were very limited in 2021 (Furcate, East Walkinshaw, and West Walkinshaw), we deployed temperature loggers for 14 days in late summer 2023. For two other sites where 2021 data were limited, we used historical stream logger data from 2012–2013 (Oshawa) and 2011–2012 (Bowmanville). Using these data, the limited 2021 data, and corresponding weather data, we created linear models in a similar way to back-calculate stream temperatures in 2021. Hereafter, we refer to all ambient stream or acclimation temperature data, whether recorded or back-calculated, as stream temperature.

### Statistical analyses

All statistical analyses were conducted in *R* for Mac OS X (R Core Team, 2022, version 4.2.0). After removing the 10 fish that did not recover from CT_max_ trials, 384 fish were included in analyses. Acclimation period was modelled to determine which interval of temperature data (in number of days) was the best predictor of CT_max_. Once the best-fit acclimation period was determined, it was used as the acclimation period in further models.

Acclimation periods were modelled using linear mixed effects models (*nlme* package; Pinheiro *et al.*, 2024) with CT_max_ as a function of a given duration of acclimation (fixed effect; 1-day, 4-day, 8-day, 14-day, 30-day, or 40-day mean temperature) and site (random effect). Only a single acclimation period was included in each candidate model given the likelihood of a high degree of correlation between overlapping acclimation periods at a site (i.e., We fit separate models for each acclimation duration then compared their relative fits). The best-fit model was determined using Akaike’s Information Criterion (AIC) and *R*^2^ using the *stats*, *AICcmodavg*, and *performance* packages (Mazerolle, 2020; Lüdecke *et al.*, 2021; R Core Team, 2022). We chose to use mean temperature in each period rather than other metrics (e.g., average maximum daily temperature, average diel fluctuation, etc.) because temperature metrics were highly correlated within sites (*r* > 0.7), mean temperature is the most easily reported, comparable, and repeatable for future studies, and it accounts for variable temperature logging intervals among sites.

We modelled CT_max_ using generalized additive mixed effects models (*mgcv* package; Wood, 2017) to evaluate the differences among sites that were unexplained by acclimation temperature. Candidate models included combinations of the best-fit acclimation period, fork length, and day of year as fixed effects, and site as a random effect. Use of a smoothing term on 30-day acclimation temperature did not improve model fit, so it was modelled only as a linear predictor. Given evidence that body size and life stage may have a non-linear relationship with thermal tolerance (Dahlke *et al.*, 2020), and that our study included young-of-year to reproductively mature adult fish, fork length was modelled using thin plate splines. We modelled day of year using cyclic cubic regression splines. No candidate model included both site and day of year, as each site was only sampled once and no two sites were sampled on the same day, therefore confounding the two variables. All models that included day of year as a fixed effect exhibited signs of underfitting, indicating they did not adequately capture the complexity of the data, and therefore were not included in model selection. The best-fit model was determined using AIC and *R*^2^ using the *stats*, *AICcmodavg*, and *performance* packages (Mazerolle, 2020; Lüdecke *et al.*, 2021; R Core Team, 2022). Fork length was chosen to represent body size, as fork length and mass were highly correlated across the dataset (*r* = 0.94). Site could only be included as a random intercept, and not as a random slope, given all individuals at any site had the same acclimation window, and the sample size and range of body sizes at each site were limited (causing model convergence and fit concerns), meaning we were not able to evaluate differences in the relationship between acclimation or fork length and CT_max_ within sites, only among them.

Thermal safety margins (TSMs) were defined as the difference between a population’s CT_max_ and the maximum stream temperature during the summer and early fall of the study year. TSM was calculated by subtracting the maximum stream temperature (including temperatures recorded *in situ* and predicted in back-calculations) between 1 May and 31 October 2021 from the population’s mean CT_max_.

Hereafter, we use the term ‘site’ rather than ‘population’, because there were at least two cases where between-site movement (genetic mixing) was possible. The two Pigeon River sites are *ca.* 2 km apart: Pigeon Main is the main branch upstream of the tributary site labelled Pigeon Tributary. Harper Creek and Byersville Creek are both in the Harper Creek watershed, only separated by *ca.* 1.2 km, and radio telemetry has revealed some movement throughout the watershed that differs based on life stage, body size, and season (Blair *et al.*, 2021; Gutowsky *et al.*, 2023).

## Results

Brook trout varied considerably in size across the study streams. Sampled fish varied in length from 74 to 289 mm (132 ± 38 mm; mean ± S.D.) and from 4.3 to 258.3 g (32.95 ± 33.66 g). Within-site variation in fish size was diverse across the study: the site with the largest difference was Byersville, where fish size ranged by 205 mm (fork length) and by 252.0 g (mass). At the least variable site, Pigeon Upstream, fish size ranged by only 60 mm and 24.0 g. The within-site mean body size varied from 94.06 (Oshawa) to 157.67 mm (East Holland).

### Critical thermal maximum

**Figure 2:**
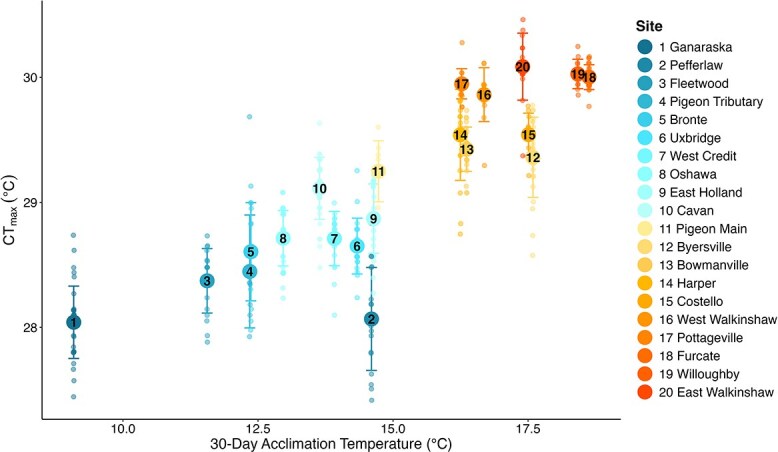
Brook trout CT_max_ and 30-day acclimation temperature (i.e., stream temperature) at 20 stream sites across Ontario. CT_max_ increased by 0.23°C for every 1°C increase in 30-day acclimation temperature. Large circles and error bars indicate site mean ± S.D. and small circles indicate individual fish (*n* = 384). Overlaid numbers correspond to legend, where sites are ordered and coloured by increasing mean CT_max_. Individual points jittered for presentation.

Acclimation temperature and site-level differences contributed to the substantial variation in CT_max_. The overall mean CT_max_ was 29.05 ± 0.69°C (mean ± S.D.), with a 3.05°C range in CT_max_ among all fish (27.41–30.46°C) (Fig. 2). Of the six acclimation periods we tested, AIC was lowest and *R*^2^ was highest in the model predicting CT_max_ with a 30-day acclimation period (Table 2). However, all acclimation periods were good predictors of CT_max_ based on *R*^2^ (all marginal *R*^2^ > 0.6, Table 2), and there were negligible differences in model fit based on ∆AIC and *R*^2^ between 40-, 30-, 14- and 4-day acclimation periods.

**Table 2:** Summary of AIC and *R*^2^ for model selection of acclimation period as a predictor of brook trout thermal maximum. Candidate general linear mixed models included one acclimation period (1-day, 4-day, 8-day, 14-day, 30-day, 40-day) as a fixed effect, and site as a random effect (random intercept; indicated by brackets). Models are listed in order of best fit based on AIC. Other listed parameters are degrees of freedom (K), cumulative AIC weight (CWt), conditional *R*^2^ and marginal *R*^2^.

**Model**	**K**	**AIC**	**ΔAIC**	**CWt**	***R*** ^**2**^ **(cond.)**	***R*** ^**2**^ **(marg.)**
1. 30-day + (Site)	4	153.02	0.00	0.52	0.868	0.659
2. 14-day + (Site)	4	155.17	2.15	0.70	0.873	0.650
3. 40-day + (Site)	4	155.23	2.20	0.87	0.862	0.617
4. 4-day + (Site)	4	156.84	3.81	0.95	0.868	0.617
5. 8-day + (Site)	4	158.65	5.62	0.98	0.872	0.598
6. 1-day + (Site)	4	159.59	6.57	1.00	0.867	0.569
7. Null	3	172.62	19.60	1.00	0.862	0.00

Including body size (fork length, mm) marginally improved the fit of the 30-day model (Table 3). Body size and 30-day acclimation temperatures explained 67% of the deviance in the data, with a further 20% explained by the random effect of site (i.e., site-level differences; deviance explained = 66.9% vs. 86.5%; Models 5 vs. 1, Table 3). For every 1°C increase in mean stream temperature during the preceding 30-day window, CT_max_ increased by 0.23°C (± 0.029°C SE), to a maximum population mean value of 30.09°C (Fig. 2). The effect of body size on CT_max_ across all sites, although detected, was negligible and nonlinear; CT_max_ increased with fork length to *ca.* 124 mm, but decreased with increasing fork length beyond the 124 mm inflection point (1.8% of deviance explained after controlling for acclimation temperature; Table 3; Supplementary S5). There was also a negative correlation (*r* = −0.64) between the mean CT_max_ of a site and range of CT_max_ values at that site, whereby variation decreased as acclimation pushed CT_max_ higher (Fig. 2). Day of year was not an accurate predictor of CT_max_, as it was consistently underfit in each candidate model (Supplementary S6).

**Table 3:** Summary of model selection for CT_max_ using the best-fit acclimation period (see Table 2). Candidate generalized additive mixed models included 30-day acclimation period and body size as fixed effects and site as a random effect (random intercept only; indicated by brackets). Models are listed in order of best fit based on AIC. Other listed parameters are degrees of freedom (df; sum of all parametric [linear] and smooth [non-linear] terms’ df), cumulative AIC weight (CWt), adjusted *R*^2^ (adj. *R*^2^), and deviance explained (Dev. Exp. %; proportion of variability in response variable explained by the model).

**Model**	**df**	**AIC**	**ΔAIC**	**CWt**	***R*** ^**2**^ **(adj.)**	**Dev. Exp. (%)**
1. 30-day + Body size + (Site)	23.13	78.05	0.00	0.55	0.857	86.5
2. Body size + (Site)	23.60	78.53	0.48	0.45	0.857	86.5
3. 30-day + (Site)	20.29	92.10	14.05	1.00	0.851	85.8
4. (Site)	20.81	92.60	14.55	1.00	0.851	85.8
5. 30-day + Body size	5.32	386.50	308.45	1.00	0.666	66.9
6. 30-day	3.00	402.41	324.36	1.00	0.65	65.1
7. Body size	9.60	784.49	706.44	1.00	0.068	8.69

### Thermal safety margins

**Figure 3:**
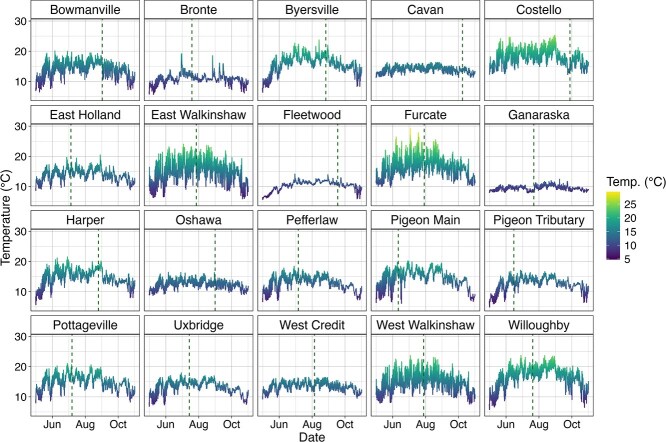
Hourly stream temperature (°C) at 20 brook trout CT_max_ sites across Ontario from 1 May 2021 to 31 October 2021. Vertical dashed lines depict site trial date. Where hourly stream temperature records were incomplete, water temperature was back-calculated (see *Materials and Methods*, Supplementary S1, S4).

**Figure 4:**
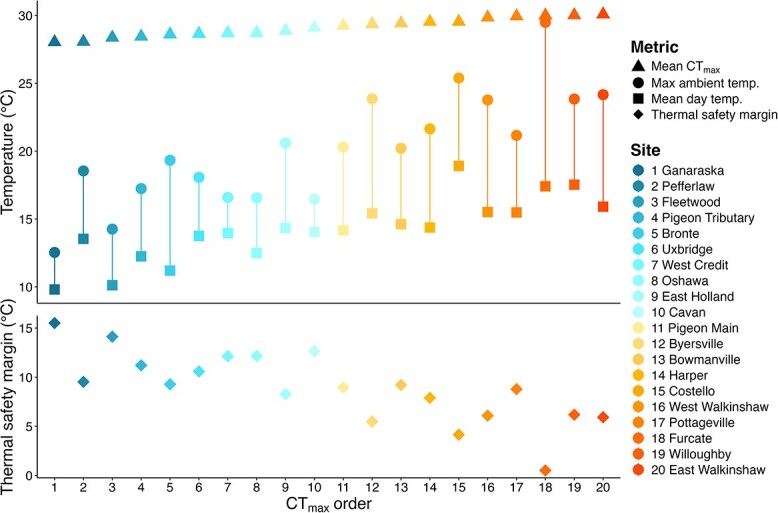
Site thermal metrics describing brook trout thermal safety margins between 1 May 2021 and 31 October 2021. Mean site CT_max_ (°C) shown as triangles, maximum stream temperature (°C) as circles, and mean daytime (0900–1900 EDT) stream temperature (°C) as squares in upper panel; thermal safety margin (difference between mean CT_max_ and maximum stream temperature, °C) as diamonds in lower panel. Sites are ordered and coloured based on increasing mean CT_max_, and correspond with numbers on *x*-axis.

Thermal regimes were markedly diverse among the 20 sites (Fig. 3). Mean stream temperatures in the month prior to CT_max_ ranged from 9.09°C (Ganaraska) to 18.63°C (Furcate) (Fig. 3, Fig. 4, Supplementary S7). Stream temperatures ranged over the season (May 1–October 31) as little as 4.75°C and as much as 19.86°C (hourly mean temperature; Ganaraska, Furcate, respectively). Average diel fluctuations were between 0.98 and 6.43°C across all sites, but the maximum diel fluctuation varied more widely: the maximum diel change was as little as 2.97°C (Ganaraska) or as much as 13.53°C (Furcate) (Supplementary S7). The absolute maximum temperature (used for calculating TSM) occurred in June for eleven sites, July for three sites, August for five sites, and September for one site (Supplementary S7). Ten of the twenty sites’ absolute maximum temperature occurred within the 30 days prior to the CT_max_ trial. The absolute maximum occurred after the CT_max_ trial at seven sites (East Holland, Ganaraska, Harper, Oshawa, Pigeon Main, Pigeon Tributary, and West Credit), and prior to the 30 days before CT_max_ at three sites (Bowmanville, Cavan, and Fleetwood) (Fig. 3, Supplementary S7).

Thermal safety margins were broadly representative of the diversity in thermal regimes among sites (Fig. 4). The highest TSM was 15°C greater than the smallest (15.51 and 0.51°C, respectively; Fig. 4). Site TSM did not rank in the same order as CT_max_: the four lowest TSMs were at Furcate, Costello, Byersville and East Walkinshaw (0.51, 4.15°C, 5.49°C, and 5.92°C, respectively), and the four highest were at Ganaraska, Fleetwood, Cavan and Oshawa (15.51, 14.12, 12.65 and 12.15°C, respectively) (Fig. 4, Supplementary S7).

## Discussion

The data obtained from our field-based CT_max_ trials highlight the contribution of acclimation and, to a lesser extent, local adaptation and other site-level factors to thermal performance of brook trout populations. Acclimation temperature explained most of the variation in CT_max_ across the dataset. Some of the remaining variation was due to site-level differences not accounted for by acclimation, which may have been influenced by local adaptation, trial date, thermal refugia, or other factors. Thermal safety margins varied widely and inconsistently with CT_max_ rank across the sites, emphasizing the importance of watershed and stream characteristics that stabilize stream temperatures. Although some studies have suggested body size as a key factor in thermal tolerance (Daufresne *et al.*, 2009; Messmer *et al.*, 2017), the effect of body size detected in our study was negligible and unlikely to be of biological significance. Together, these findings demonstrate the utility of field-based studies in quantifying intraspecific variation in thermal performance and exploring its causes.

### Acclimation, body size, and site-level effects on CT_max_

This is the first study to quantify brook trout thermal tolerance *in situ*. The CT_max_ range here was within the range of what has been reported in laboratory work (between 26 and 31.9°C) in subadult and adult brook trout (Lee and Rinne, 1980; Benfey *et al.*, 1997; Galbreath *et al.*, 2004; McDermid *et al.*, 2012; Stitt *et al.*, 2014; Wells *et al.*, 2016; Morrison *et al.*, 2020; O’Donnell *et al.*, 2020; Mackey *et al.*, 2021; Stewart *et al.*, 2023) and brook trout at our sites were acclimated to a range of temperatures captured within with those tested *ex situ*. Acclimation metrics (temperature and duration of acclimation; between 5 and 25°C) and CT_max_ methodologies, including warming rates (between 2 and 18°C h^−1^) and procedures (e.g., Elliot’s hybrid CT_max_ in McDermid *et al.*, 2012 and Stitt *et al.*, 2014), have varied widely across previous studies (see above references). The relationship between acclimation and CT_max_ in our study (+ 0.23°C per 1°C increase in 30-day acclimation temperature) scales similarly to previous work on brook trout (+ 0.26°C per 1°C increase between 5 and 25°C in Morrison *et al.*, 2020; + 0.35°C per 1°C increase between *ca.* 11.03 and 16.83°C in Stewart *et al.*, 2023) and to a meta-analysis including 68 species of fish (+ 0.25°C per 1°C increase in acclimation temperature across 68 fish species from 83 studies in Pottier *et al.*, 2022).

Beyond the effects of acclimation, body size (fork length) had a negligible but detectable effect on CT_max_ across our dataset after accounting for differences in stream temperature among sites. CT_max_ was highest at intermediate body sizes and was lowest at the largest, aligning with literature that suggests thermal tolerance varies with life stage (see meta-analysis in Dahlke *et al.*, 2020), and tends to decline with increasing body size (see review in McKenzie *et al.*, 2021). No recent studies of brook trout thermal tolerance have reported notable body size effects, but all have been laboratory-based and used a single age class (see above references). Be that as it may, further research is needed to clarify the effect detected here, as limitations in our study, such as the exclusion of small young-of-year fish and suspected within-site variation in acclimation temperatures (see below), could be related to the observed body size effect.

Much of the remaining variation in CT_max_ was attributed to site-level effects. Local adaptation (genetic differences) could have contributed to some of this variation, although common garden experiments would be required to clarify genetic effects. McDermid *et al.* (2012), Stitt *et al.* (2014), Cook *et al.* (2018) and Stewart *et al.* (2024) all found evidence supporting local adaptation and genetic effects on thermal tolerance in Ontario brook trout under common garden conditions. By contrast, Wells *et al.* (2016) did not find this among six Newfoundland populations, although it should be noted that their populations shared common ancestry and were from a smaller geographical area than the other studies. Other site-level differences may have also contributed to site being a significant predictor of CT_max_ after controlling for acclimation temperature. For instance, trial arena acclimatization times varied within and among sites as a result of collection procedures and limitations, introducing variable lengths of recovery time post-handling and electrofishing. Trial date was another factor that we were unable to account for in our study; each site could only be measured once, and only one site could be measured on a given day. A population’s CT_max_ can vary with seasonal changes in thermal acclimation (Turko *et al.*, 2020; Reemeyer *et al.*, 2024), so we suspect that some sites’ CT_max_ was lower than if we had tested it later in the season after prolonged acclimation to warm temperatures, as only half of our sites’ maximum stream temperatures occurred in the 30 days prior to the CT_max_ trial. In addition to seasonal acclimation, we suspect that individual-level differences in acclimation could exist within sites. Brook trout thermoregulate using groundwater upwellings and other thermal refugia (e.g., shaded pools, coldwater tributaries), particularly when ambient temperatures reach above a given threshold (Baird and Krueger, 2003; Petty *et al.*, 2012; Goyer *et al.*, 2014; Hitt *et al.*, 2017; White *et al.*, 2019). Access to and use of groundwater upwellings for behavioural thermoregulation varied across sites in our study. We observed brook trout aggregating in thermal refugia at some sites; thus, our mean stream temperature values likely differed somewhat from the mean body temperature of fish that were using distinct thermal microhabitats. As a result, individual acclimation temperatures could have varied, potentially influencing the degree of variation in CT_max_ at a single site or between sites with similar 30-day acclimation temperatures (e.g., Pefferlaw and East Holland sites).

Acclimation effects on brook trout CT_max_ have previously been assessed in the laboratory. Morrison *et al.* (2020) found that brook trout CT_max_ did not increase with acclimation beyond 20°C, and their study reported the highest recorded CT_max_ for brook trout at 31.7 and 31.9°C (no significant difference between groups acclimated to 20 and 23°C). Interestingly, the next two highest reported brook trout CT_max_ values were from fish acclimated to *ca.* 17°C for 30 days (mean = 29.94°C; Stewart *et al.*, 2023) and this study, where the highest site mean CT_max_ was 30.09°C with a 30-day acclimation period mean of 17.44°C. These two values were both recorded under a slower warming rate than in Morrison *et al.* (2020), who used 0.3°C min^−1^ (18°C h^−1^) vs. 4°C h^−1^ in the present study. In general, a lower CT_max_ should be expected with a slower warming rate because the duration of time spent above a critical temperature is longer, thereby accumulating more thermal injury (Ørsted *et al.*, 2022). Together, these studies support the idea of a ‘ceiling’ to upper thermal tolerance where positive effects of acclimation to higher temperatures plateau, coinciding with an acclimation temperature where physiological performance begins to be limited (i.e., a supra-optimal temperature) (Sandblom *et al.*, 2016; Morrison *et al.*, 2020).

### Sublethal thresholds and thermal risk

There are multiple lines of evidence that indicate wild brook trout populations may occupy habitats with seasonally supra-optimal temperatures and incur sublethal effects as a result. In brook trout, growth declines above 16°C and negative growth occurs above 23.4°C (Chadwick and McCormick, 2017), aerobic scope decreases above 15°C (Durhack *et al.*, 2021), acclimation becomes limited above 20°C (Mackey *et al.*, 2021), and systematic cellular stress becomes pronounced around 22°C (Lund *et al.*, 2003; Chadwick *et al.*, 2015; Chadwick and McCormick, 2017; Mackey *et al.*, 2021). In lakes, air and water temperatures above 20–22°C affect brook trout reproductive timing and success (Robinson *et al.*, 2010; Warren *et al.*, 2012), and they change their behavioural tactics when epilimnion temperatures reach 22.4°C (Goyer *et al.*, 2014). Brook trout populations decline or are absent when stream temperatures are above 20–24°C (Creaser, 1930; Barton *et al.*, 1985; Meisner, 1990; Eaton *et al.*, 1995; Martin and Petty, 2009; Petty *et al.*, 2012; DeWeber and Wagner, 2015). Collectively, these studies indicate *ca.* 22.5°C as a likely sublethal threshold for brook trout. Here we observed (in raw or back-calculated data) temperatures exceeding the optimum range of brook trout (>20°C) in 11 of 20 sites across the season (1 May–31 October); six of these include temperatures above the sublethal physiological threshold (> 22.5°C). We are unsure, however, if the duration of exposure to supra-optimal temperatures was enough for negative effects on fitness. If we consider the sublethal threshold for TSMs instead of using CT_max_, then six of our sites had a negative TSM, meaning the fish there are likely already physiologically stressed by warm water at some points during the summer. Accordingly, assessments of the risk posed by warming should consider not only lethal limits, but the sublethal temperatures at which fitness begins to decline (Pinsky *et al.*, 2019; Mayer *et al.*, 2024).

Plasticity in thermal tolerance can be quantified using the acclimation response ratio (ARR), which describes the change in thermal tolerance relative to the change in acclimation temperature (Claussen, 1977; Gunderson and Stillman, 2015; Ruthsatz *et al.*, 2024). We saw only a small increase in CT_max_ in response to a *ca.* 9°C increase in acclimation. As a result, the ARR here was less than 1, consistent with other literature on brook trout (see above). CT_max_ increased at one-fifth the rate that TSM decreased; thus, the plasticity of upper thermal tolerance in brook trout is unlikely to be sufficient for reducing physiological risks from global warming (Gunderson and Stillman, 2015; McArley *et al.*, 2017; Pottier *et al.*, 2022). That said, the duration, temperature, and isothermality of acclimation, as well as the CT_max_ ramping rate, can influence the ARR (Ruthsatz *et al.*, 2024). The same ARR phenomenon has been reported in both marine and freshwater fishes: increased acclimation temperatures resulted in a CT_max_ plateau and a decreasing TSM in common threefin *Forsterygion lapillum* (McArley *et al.*, 2017), redside dace *Clinostomus elongatus* (Turko *et al.*, 2020), and eastern sand darter *Ammocrypta pellucida* (Firth *et al.*, 2021). Reversible plasticity (i.e., acclimation) is likely insufficient for buffering the physiological effects of warming on brook trout in the long-term, and consequently, the biophysical characteristics of streams and watersheds are key to defining the risk and resilience of populations.

### Determinants of risk in brook trout watersheds

Our research was conducted at sites representing a range of lotic brook trout habitats in Ontario, including forested groundwater-dominated streams, lake-fed bedrock creeks, and creeks flowing through agricultural and urban areas. The wide range of TSMs and exposure to supra-optimal temperatures across the twenty sites points to the importance of conservation and management at the population level. Regional, watershed, and site-specific characteristics like watershed land cover, tributary confluences, groundwater upwellings, riparian shading, and deep pools are key determinants of thermal habitat suitability for brook trout and other cold- and cool-water fishes (Siitari *et al.*, 2011; Petty *et al.*, 2012; Argent and Kimmel, 2013; Gallagher and Fraser, 2024). Of our six sites with the lowest TSMs, four were creeks that were fed by lakes and had a high degree of watershed connectivity (Furcate, Costello, East Walkinshaw, West Walkinshaw). Although being fed by lake surface water can cause a great deal of warming in summer months (Mellina *et al.*, 2002; Chu *et al.*, 2010), it is likely that brook trout in these creeks migrate to access coldwater refugia, either to discrete groundwater upwellings in the creek (Curry and O’Sullivan, 2023) or into stratified lakes, as a means of behavioural thermoregulation, thus limiting their exposure to suboptimal or sublethal temperatures (Fraser and Bernatchez, 2008). Brook trout are known to move within watersheds to reach suitable thermal habitat, whether for growth, spawning, or refuge (Stranko *et al.*, 2008; Petty *et al.*, 2012; Blair *et al.*, 2021; Gutowsky *et al.*, 2023). Aside from the lake-fed creeks, the three sites with the lowest TSMs included two creeks whose watersheds are heavily urbanized (Byersville and Harper, both of which are in the city of Peterborough, Ontario) and a third whose upstream catchment is primarily cleared agricultural land (Willoughby). The lack of permeability and/or riparian shading in these catchments is likely contributing to warmer average and maximum temperatures. Even low levels of urban and agricultural development can contribute to increased impervious cover, channelization, or loss of riparian shading, which in turn can affect brook trout through changes to stream temperature, morphology, and habitat (Wang *et al.*, 2003; Stanfield and Kilgour, 2006; Stranko *et al.*, 2008; DeWeber and Wagner, 2015).

Heat waves already pose a risk to brook trout in habitats where average summer temperatures approach the supra-optimal physiological threshold (*ca*. 22.5°C), as heat waves there reach or surpass the sublethal, or even lethal, threshold (Gunn and Snucins, 2010). Although plasticity is able to buffer some of the effects of warming, the ‘concrete ceiling’ of upper thermal tolerance remains (Sandblom *et al.*, 2016). Plastic responses are complex across varying durations and intensities of thermal stress (Rezende *et al.*, 2014), and the increasing frequency and severity of heat waves may go beyond the capacity of organisms to recover (Dai *et al.*, 2022). Streams with more groundwater input are less sensitive to air temperature changes and thus resilient to climate change (Chu *et al.*, 2008; Siitari *et al.*, 2011; Argent and Kimmel, 2013; Gallagher and Fraser, 2024; Kelly and Kelly, 2024). In our study, the streams with the highest TSMs were the least developed and had groundwater influence from the Oak Ridges Moraine and Niagara Escarpment. Groundwater-mediated streams are vital refugia for brook trout, and the identification and protection of these refugia are important conservation measures (Chu *et al.*, 2008; Isaak and Young, 2023; Mejia *et al.*, 2023). Ultimately, higher temperatures put brook trout at risk of extirpation, but site-specific factors must be considered when prioritizing conservation actions (Childress *et al.*, 2024).

### Summary and future directions

Our findings suggest that field-based methodologies can be used to build on evidence gathered in lab-based studies and explore the mechanisms behind thermal tolerance in fishes. We have provided evidence that the effect of acclimation on upper thermal tolerance *ex situ* is reflected *in situ*, that watershed characteristics are a key driver of intraspecific variation, and that site-level effects beyond temperature contribute to the variation as well, emphasizing the need for conservation and management at or below the population level. Study designs like ours that include local thermal conditions are beneficial for synthesizing the effects of acclimation with natural variability and revealing defining characteristics related to risk.

Some have argued that CT_max_ is not ecologically relevant given the rapid warming rate and likelihood of environments reaching critical temperatures (Desforges *et al.*, 2023; Mayer *et al.*, 2024). We counter that, contrary to this interpretation, CT_max_ provides a valuable index of relative tolerance and plasticity, particularly when it is used for comparisons such as among populations or life stages, which in turn contributes to understanding the ecological consequences of warming. While comparative lab-based studies are needed to confirm local adaptation and separate heritable vs. environmental influences on CT_max_, field-based CT_max_ is more biologically representative for local populations. These data are important for identifying thermal limits and the likelihood of local population persistence under changing environmental conditions.

## Supplementary Material

Web_Material_coae086

## Data Availability

The data and code underlying this article are available on Figshare at https://doi.org/10.6084/m9.figshare.26036293 and 10.6084/m9.figshare.27600270
