# Supplementary material for: Local conditions drive interpopulation variation in field-based critical thermal maximum of brook trout
Source: Conserv Physiol. 2024 Dec 26;12(1):coae086. doi: 10.1093/conphys/coae086 (PMC11959187; doi:10.1093/conphys/coae086)
Supplement: Web_Material_coae086 [file web_material_coae086.pdf]

Local conditions drive among-population variation in field-based critical thermal maximum (CT_{max}) of brook trout

Erin M.C. Stewart, Jacob C. Bowman, Chris C. Wilson, and Graham D. Raby

Supplementary Information

S1 Ambient stream temperature data logging information from 20 brook trout CT_{max} sites

S2 Brook trout field critical thermal maximum (CT_{max}) mobile laboratory

S3 Trial arena warming rates at 20 brook trout CT_{max} sites

S4 Regression terms for back-calculations of stream temperature

S5 Overall effect of body size on CT_{max}.

S6 Smoothed effect of sample day from candidate GAM to model seasonal effects of acclimation across dataset.

S7 Summary of brook trout field CT_{max} and acclimation data from 20 sites

S1 Ambient stream temperature data logging information from 20 brook trout CT_{max} sites across Ontario, Canada. Data loggers were deployed and managed by various agencies and partners due to the active use of multiple sites as long-term study sites or for other research (CLOCA = Central Lake Ontario Conservation Authority, CH = Conservation Halton, LSRCA = Lake Simcoe Region Conservation Authority, OMNRF = Ontario Ministry of Natural Resources, GRCA = Ganaraska Region Conservation Authority, CVC = Credit Valley Conservation). 14 and 30 refer to the number of days before the site's trial date, where a dot indicates that temperature was logged during that entire period. Logger type indicated if known. Days missing indicates the number of days between May 1 – October 31, 2021 that ambient stream temperature data was not logged in 2021 (total days in season = 183). Additional temperature data was logged in 2023 at East Walkinshaw, West Walkinshaw, and Furcate for use in back-calculations, and additional historical data was used for Oshawa and Bowmanville back-calculations (see *Methods*).

Site	14	30	Logger	Logging interval	Deployed	Days missing
Bowmanville	·		RBR Solo^3	30 min	Authors	164
			—	30 min 2011, 2012	CLOCA	—
Bronte	·	·	HOBO Pendant	30 min	CH	44
Byersville	·	·	RBR Solo^3	10 min	Authors	12
Cavan	·		RBR Solo^3	30 min	Authors	146
Costello	·	·	RBR Solo^3	30 min	Authors	111
East Holland	·		—	15 min	LSRCA	37
East Walkinshaw			—	30 min 2021	OMNR	181
			RBR Solo^3	15 min 2023	E. Hegge	—
Fleetwood	·	·	RBR Solo^3	5 min	Authors	12
Furcate			—	30 min 2021	OMNR	181
			RBR Solo^3	15 min 2023	E. Hegge	—
Ganaraska	·		HOBO Pendant	15 min	GRCA	95
Harper	·	·	RBR Solo^3	10 min	Authors	12
Oshawa	·		RBR Solo^3	30 min	Authors	165
			—	30 min 2012, 2013	CLOCA	—
Pefferlaw	·		—	15 min	LSRCA	37
Pigeon Main			RBR Solo^3	10 min	Authors	38
Pigeon Tributary	·	·	RBR Solo^3	5 min	Authors	12
Pottageville	·	·	—	15 min	LSRCA	37
Uxbridge	·	·	—	15 min	LSRCA	37
West Credit	·	·	—	30 min	CVC	43
West Walkinshaw			—	NA 2021	OMNR	183
			RBR Solo^3	15 min 2023	E. Hegge	—
Willoughby	·	·	HOBO Pendant	30 min	CH	37

S2 Brook trout field critical thermal maximum (CT_{max}) mobile laboratory. *Top left:* Fish underwent CT_{max} trials in the larger of the two tanks (white cooler), which was connected to a sump tank (blue cooler in right side of photo) that housed heaters, pumps, and a large air stone. Heated and aerated water was recirculated between the two tanks continuously throughout. Upon loss of equilibrium, fish were removed from the trial arena using hand nets and put in individual tanks (buckets) to recover in cool, aerated water. Anaesthetic bath and measurement equipment on truck bed. *Top right:* Sump tank from above showing connection to CT_{max} arena and example of equipment. Actual heating rods and pumps used in mobile laboratory differed from those shown in this photo. *Bottom:* Fish were separated from large pump fittings using a permeable barrier. Water flowed into the arena from one port on either side of the cooler, and returned to the sump through two ports behind the permeable barrier. Temperature was logged in the trial arena using a Bluetooth temperature logger (black cord in photo). Lid was left closed with a 2 cm opening for air flow until fish neared CT_{max} and needed constant observation.

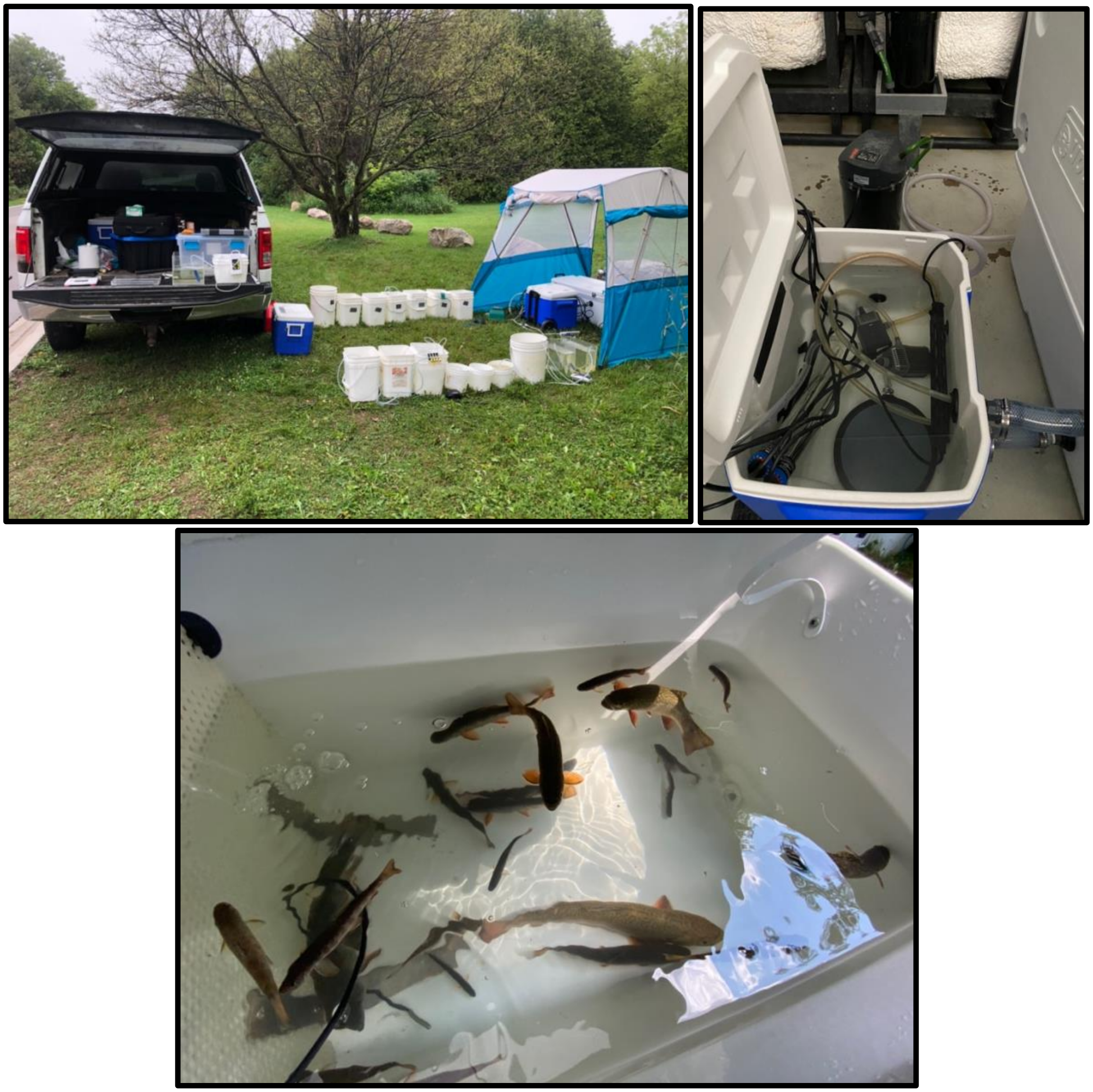

S3 Trial arena warming rates at 20 brook trout CT_{max} sites across Ontario, Canada. Trial arena tank temperature (°C) shown against time elapsed (minutes), with calculated warming rate (°C $hour^{-1}$) in bottom right corner of each box. Trials began close to ambient stream temperature and were to be warmed at a rate of 4 °C $hour^{-1}$. Trial temperatures shown from the start of the trial (minute 0, i.e., onset of heating) to when the final individual fish at the site was removed. Trials were between *ca.* 180–240 minutes based on the start temperature and CT_{max} achieved.

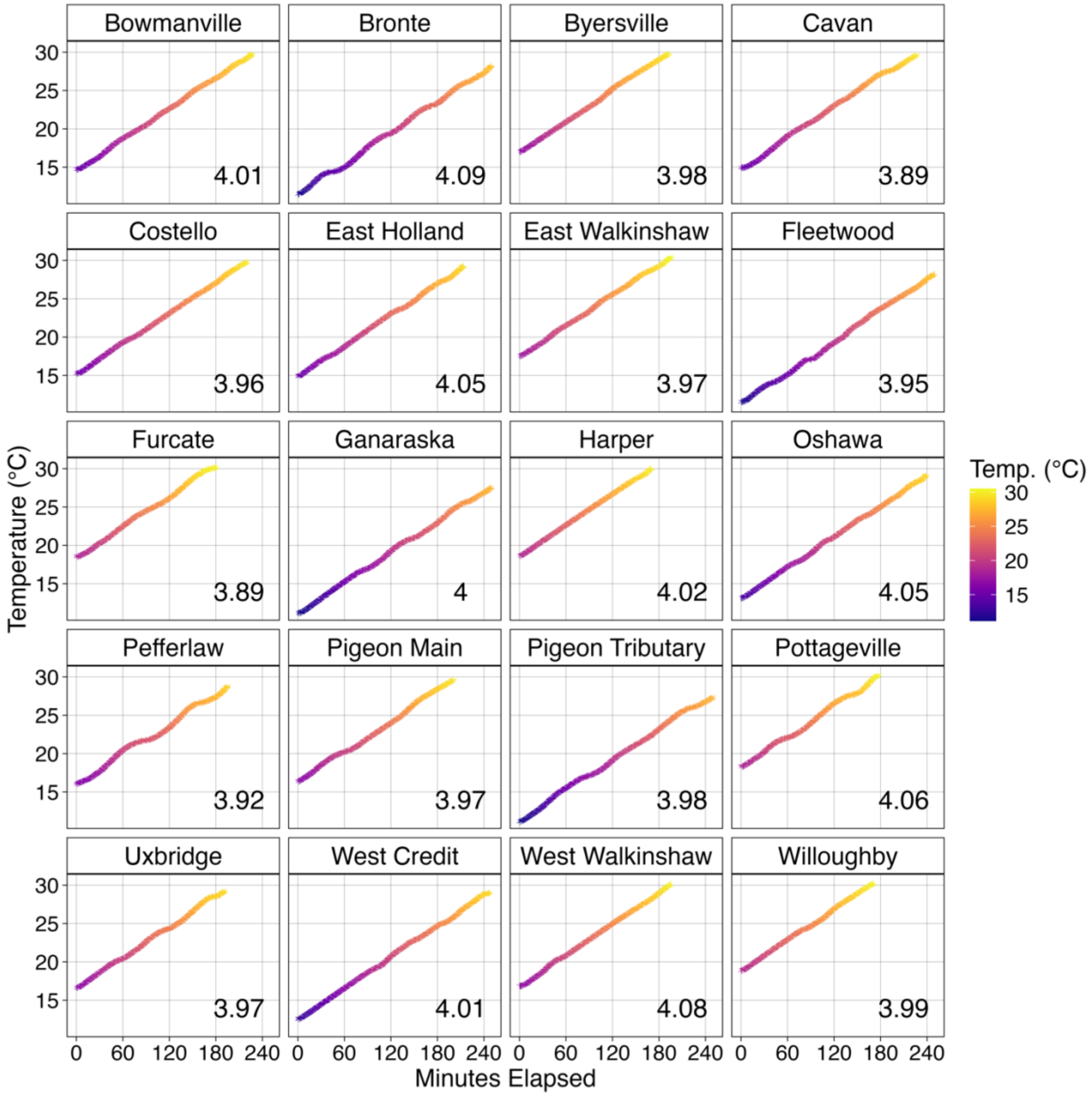

S4.a Regression terms for back-calculations of stream temperature at 20 sites across Ontario, Canada. Terms for both linear and logistic regressions are provided. R^2 values for linear and logistic predicted data show how well the predicted temperatures explained the actual observed temperature logger data. Model used is the model type used for predicting acclimation temperatures in each stream. Logger days indicates the number of days of real temperature data that were available to use for imputation with air temperatures from the indicated weather station. Stream temperature (temp.) lag describes the best time lag (hours) in stream temperature explained by variation in air temperature.

	Logistic Models			Linear Models		Residual S.D.		Observed temp ~ prediction R^2				
**Site**	**L**	**k**	**x0**	**Slope**	**Intercept**	**Logistic**	**Linear**	**Logistic**	**Linear**	**Model Used**	**Logger days**	**Stream temp. lag (hours)**
Bowmanville	20.60	0.10	9.39	0.44	6.46	1.41	1.46	0.56	0.59	Linear	160.4	5
Bronte	12.44	0.13	–0.19	0.17	7.92	1.17	1.23	0.51	0.45	Logistic	139.4	4
Byersville	21.16	0.091	4.018	0.35	9.94	1.84	1.87	0.59	0.58	Logistic	171	9
Cavan	149.23	0.013	189.78	0.16	11.25	0.54	0.54	0.57	0.57	Logistic	14	6
Costello	859.93	0.020	202.66	0.34	13.01	2.16	2.18	0.41	0.40	Logistic	31.5	4
East Holland	23.21	0.053	9.23	0.29	9.07	1.00	1.00	0.64	0.64	Logistic	128.8	4
East Walkinshaw	29.80	0.075	14.79	0.52	7.17	1.74	1.75	N/A	N/A	Logistic	17.8	4
Fleetwood	11.83	0.13	–0.72	0.15	7.84	1.14	1.19	0.44	0.39	Logistic	171.6	11
Furcate	1207.76	0.031	152.64	0.50	9.14	1.88	1.94	0.85	0.84	Logistic	15.7	3
Ganaraska	248.16	0.0098	345.02	0.090	7.95	0.73	0.73	0.32	0.31	Logistic	87	2
Harper	21.14	0.079	5.70	0.34	8.97	1.48	1.49	0.68	0.67	Logistic	171	8
Oshawa	131.67	0.023	116.92	0.25	8.15	1.50	1.51	0.59	0.59	Logistic	186.5	6
Pefferlaw	20.32	0.073	8.46	0.33	7.50	0.95	0.95	0.79	0.78	Logistic	143.9	4
Pigeon Main	20.60	0.097	6.79	0.39	8.10	1.52	1.55	0.71	0.70	Logistic	145.8	8
Pigeon Tributary	16.61	0.098	4.09	0.29	7.81	1.18	1.53	0.70	0.68	Logistic	171	8
Pottageville	24.87	0.067	10.66	0.38	8.53	1.11	1.11	0.72	0.71	Logistic	127.9	4
Uxbridge	18.66	5.077	4.018	0.31	8.24	0.88	0.90	0.79	0.78	Logistic	143.8	4
West Credit	27.74	0.033	18.30	0.23	9.75	0.90	0.90	0.51	0.52	Linear	18.5	3
West Walkinshaw	40.20	0.048	26.52	0.44	8.11	2.46	2.46	0.84	0.83	Logistic	14	4
Willoughby	23.18	0.12	9.50	0.51	7.27	1.45	1.54	0.83	0.80	Logistic	139.5	4

S4.b Environment and Climate Change Canada weather station used for air temperature data at each study site (accessed via https://climate.weather.gc.ca/index_e.html).

Site	Weather station	Distance from station (km)
Bowmanville	Oshawa	16.57
Bronte	Guelph Turfgrass	16.66
Byersville	Peterborough A	6.36
Cavan	Peterborough A	17.31
Costello	Algonquin Park East Gate	11.07
East Holland	King City North	10.49
East Walkinshaw	Thunder Bay	36.92
Fleetwood	Peterborough A	21.68
Furcate	Cameron Falls (Aut)	40.47
Ganaraska	Peterborough A	20.77
Harper	Peterborough A	4.87
Oshawa	Oshawa	9.62
Pefferlaw	Uxbridge West	5.17
Pigeon Main	Peterborough A	28.91
Pigeon Tributary	Peterborough A	27.05
Pottageville	King City North	4.46
Uxbridge	Uxbridge West	3.9
West Credit	Mono Centre	25.3
West Walkinshaw	Thunder Bay	35.7
Willoughby	Hamilton RBG Cs	12.96

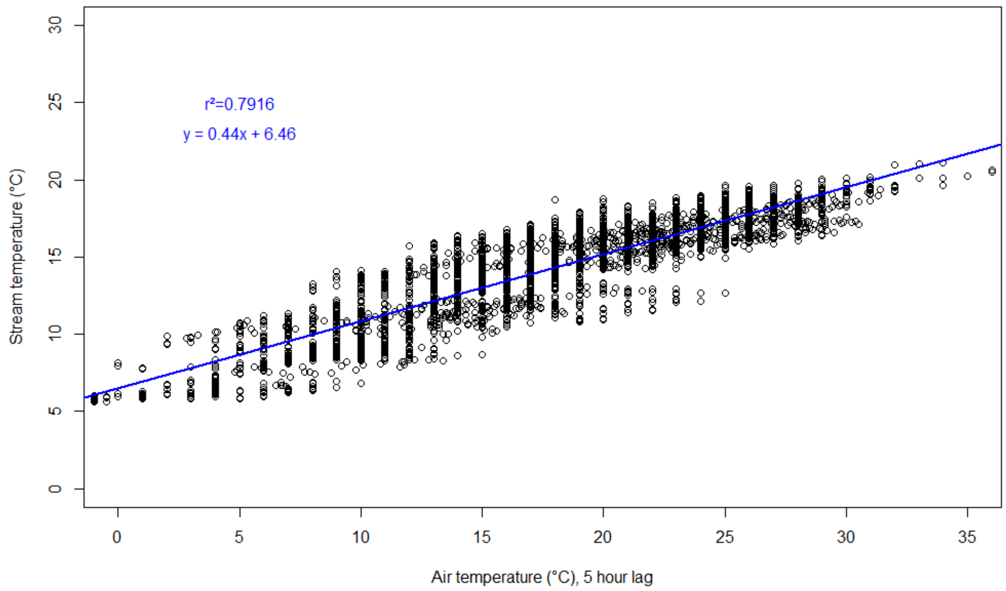

S4.c The linear relationship used to back-calculate stream temperature from air temperature data for Bowmanville Creek. Temperature data used to build this relationship were from 2011, 2012, and 2021. Variation in stream temperature was best explained by the air temperature five hours prior.

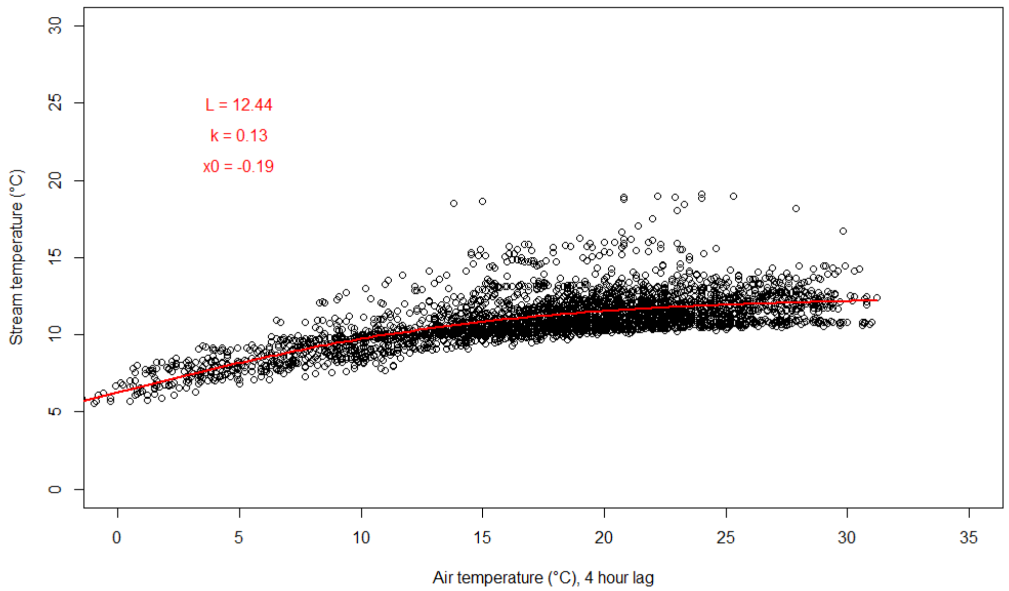

S4.d The logistic relationship used to back-calculate stream temperature from air temperature data for Bronte Creek. Temperature data used to build this relationship were from 2021. Variation in stream temperature was best explained by the air temperature four hours prior.

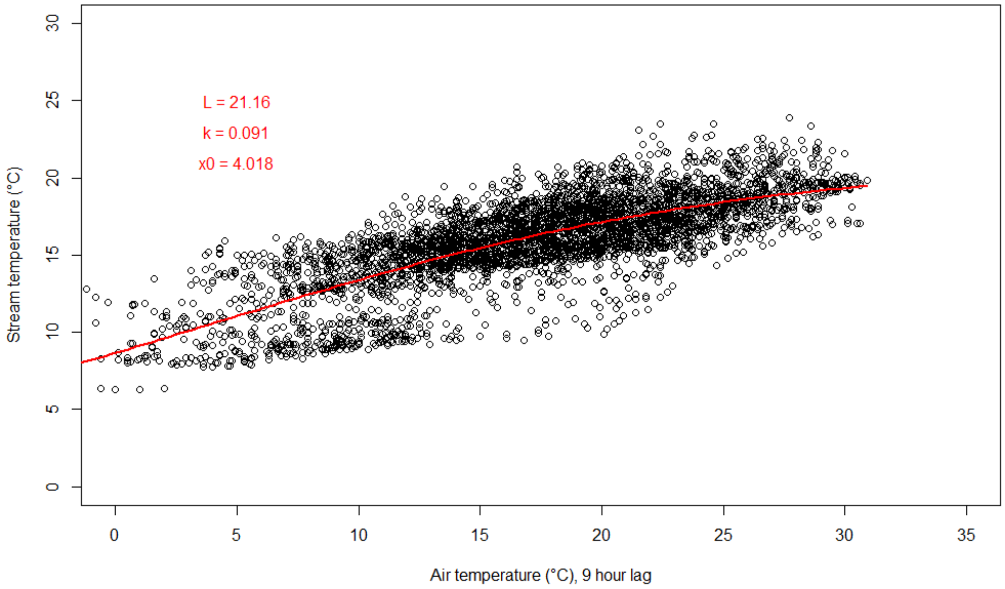

S4.e The logistic relationship used to back-calculate stream temperature from air temperature data for Byersville Creek. Temperature data used to build this relationship were from 2021. Variation in stream temperature was best explained by the air temperature nine hours prior.

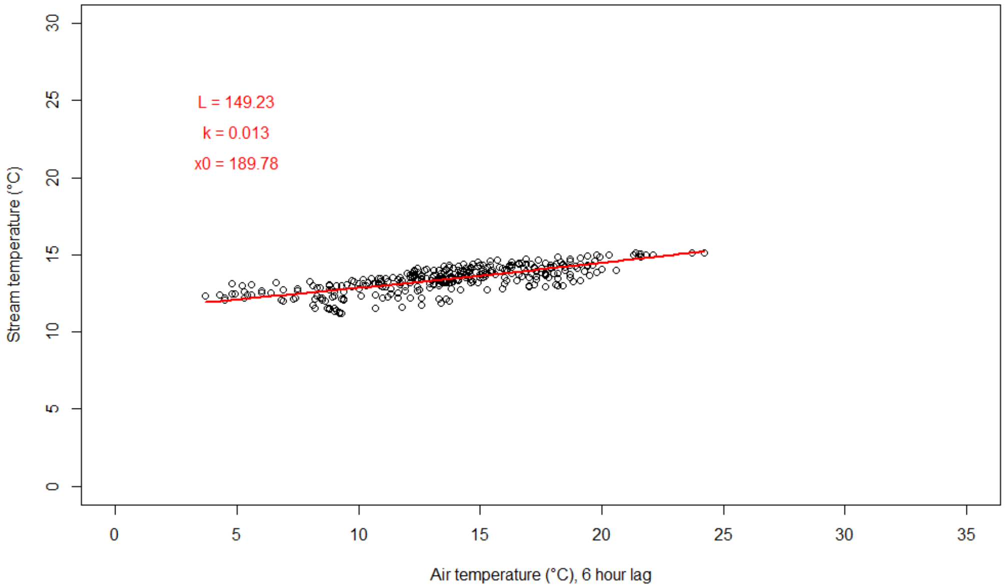

S4.f The logistic relationship used to back-calculate stream temperature from air temperature data for Cavan Creek. Temperature data used to build this relationship were from 2021. Variation in stream temperature was best explained by the air temperature six hours prior.

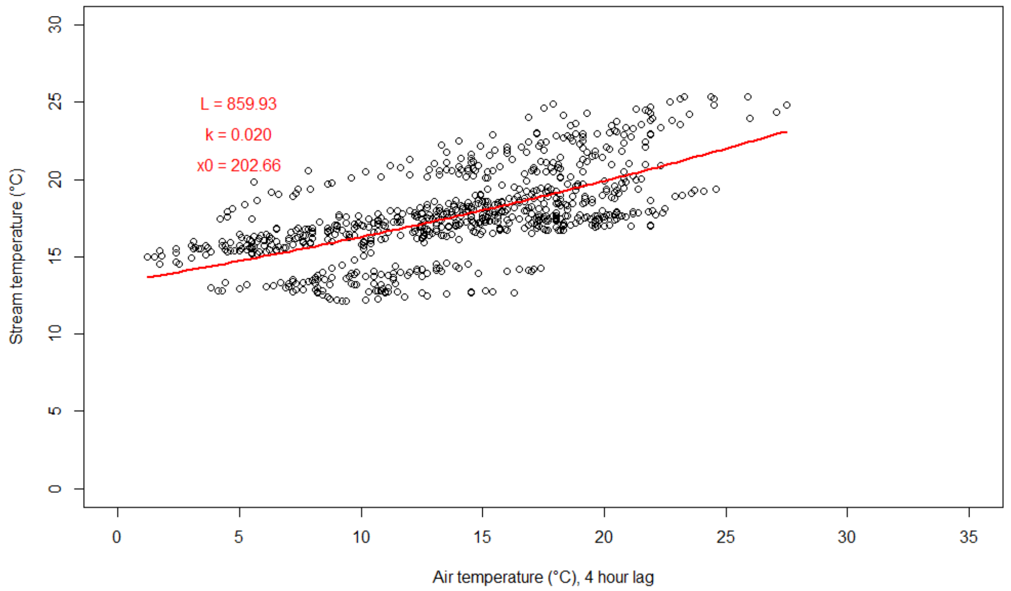

S4.g The logistic relationship used to back-calculate stream temperature from air temperature data for Costello Creek. Temperature data used to build this relationship were from 2021. Variation in stream temperature was best explained by the air temperature four hours prior.

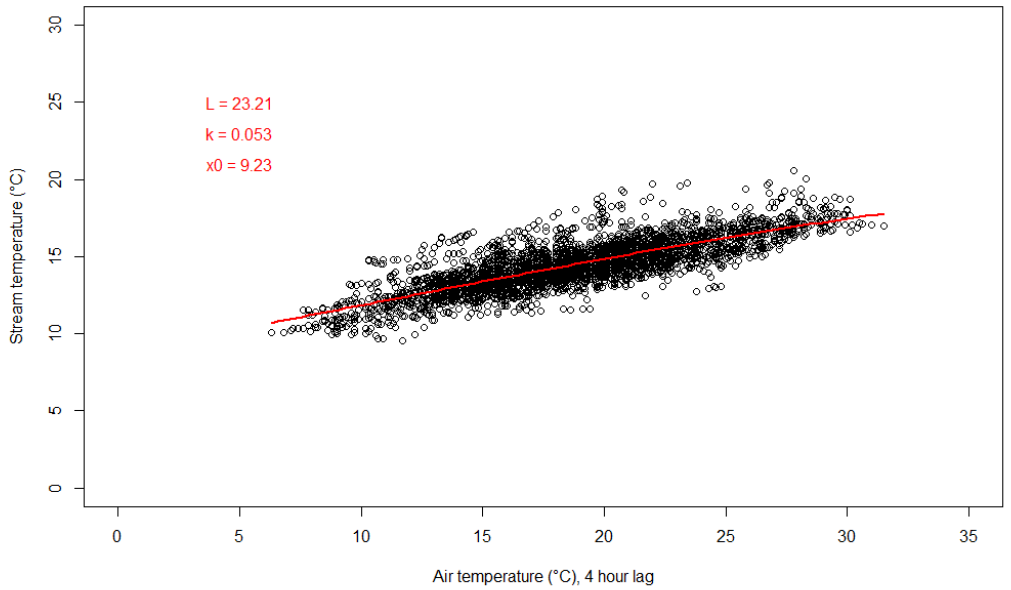

S4.h The logistic relationship used to back-calculate stream temperature from air temperature data for the East Holland River. Temperature data used to build this relationship were from 2021. Variation in stream temperature was best explained by the air temperature four hours prior.

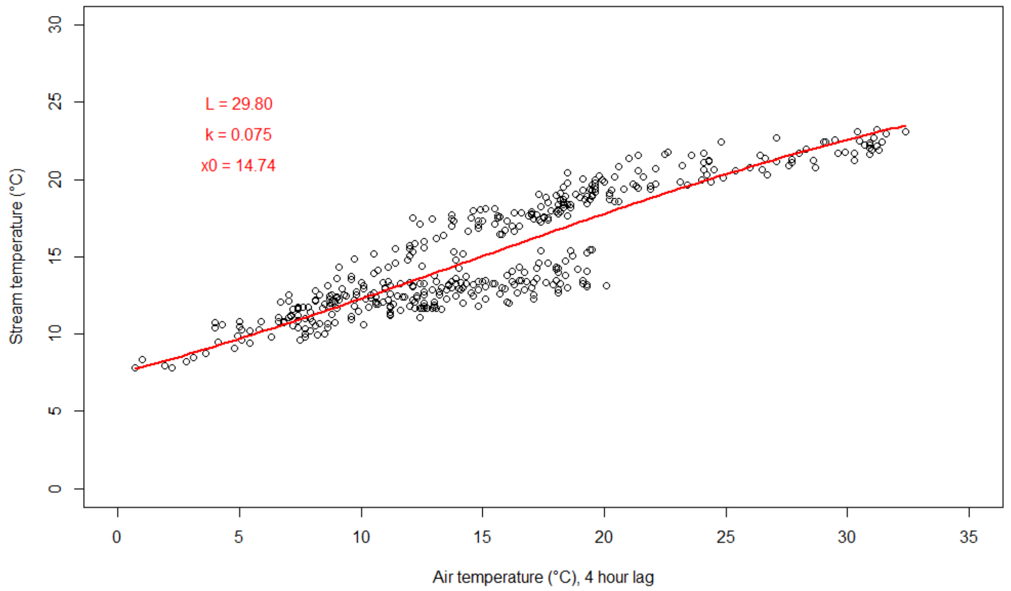

S4.i The logistic relationship used to back-calculate stream temperature from air temperature data for East Walkinshaw Creek. Temperature data used to build this relationship were from 2021 and 2023. Variation in stream temperature was best explained by the air temperature four hours prior.

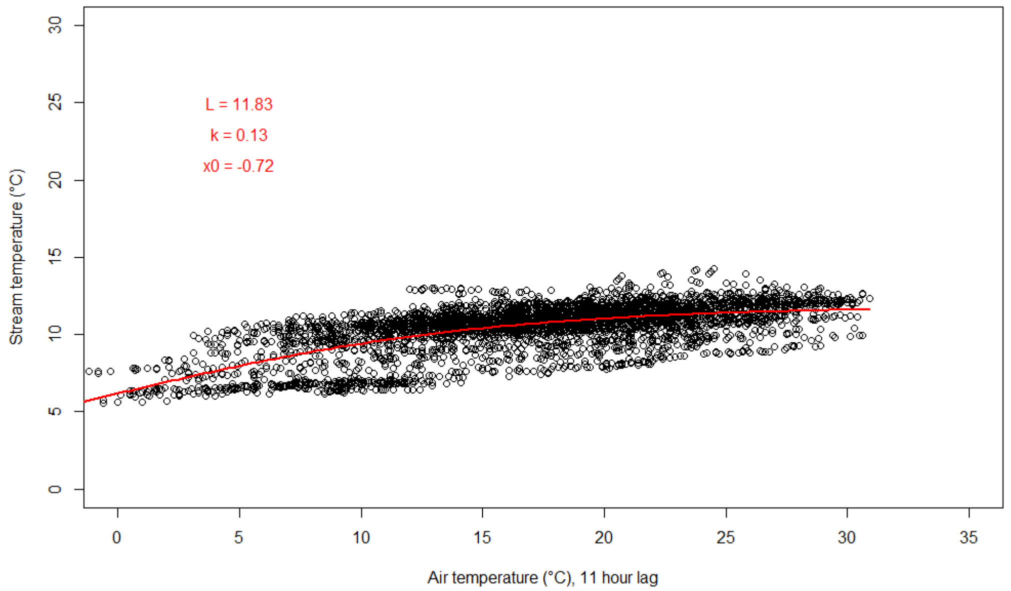

S4.j The logistic relationship used to back-calculate stream temperature from air temperature data for Fleetwood Creek. Temperature data used to build this relationship were from 2021. Variation in stream temperature was best explained by the air temperature 11 hours prior.

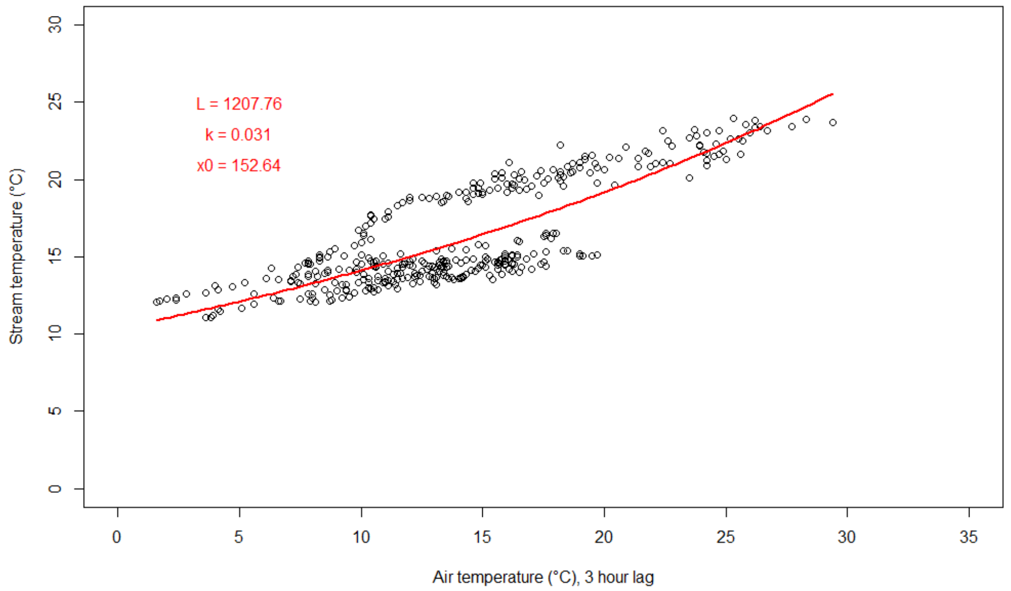

S4.k The logistic relationship used to back-calculate stream temperature from air temperature data for Furcate Creek. Temperature data used to build this relationship were from 2021 and 2023. Variation in stream temperature was best explained by the air temperature three hours prior.

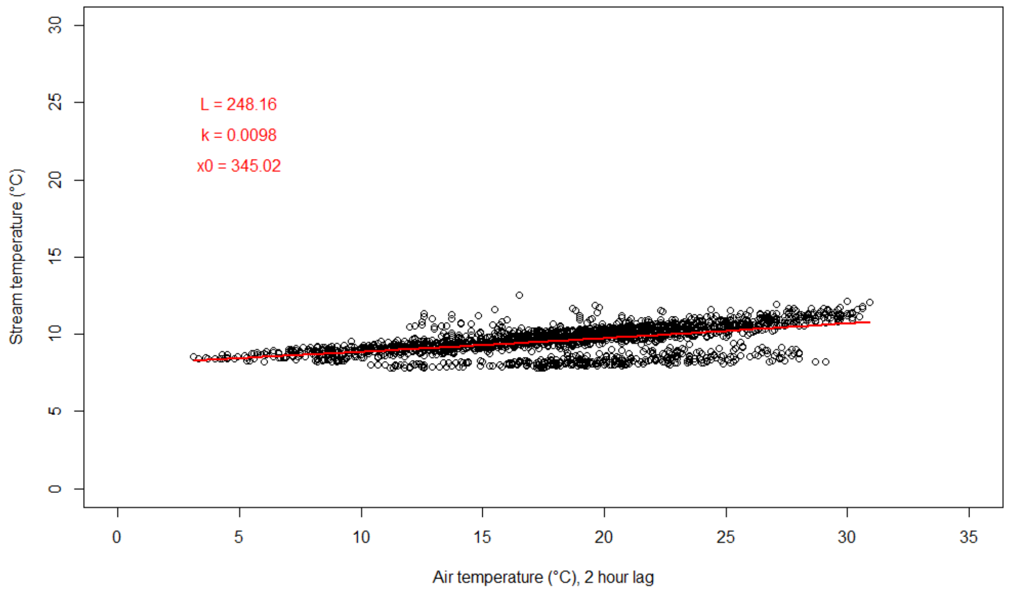

S4.l The logistic relationship used to back-calculate stream temperature from air temperature data for the Ganaraska River. Temperature data used to build this relationship were from 2021. Variation in stream temperature was best explained by the air temperature two hours prior.

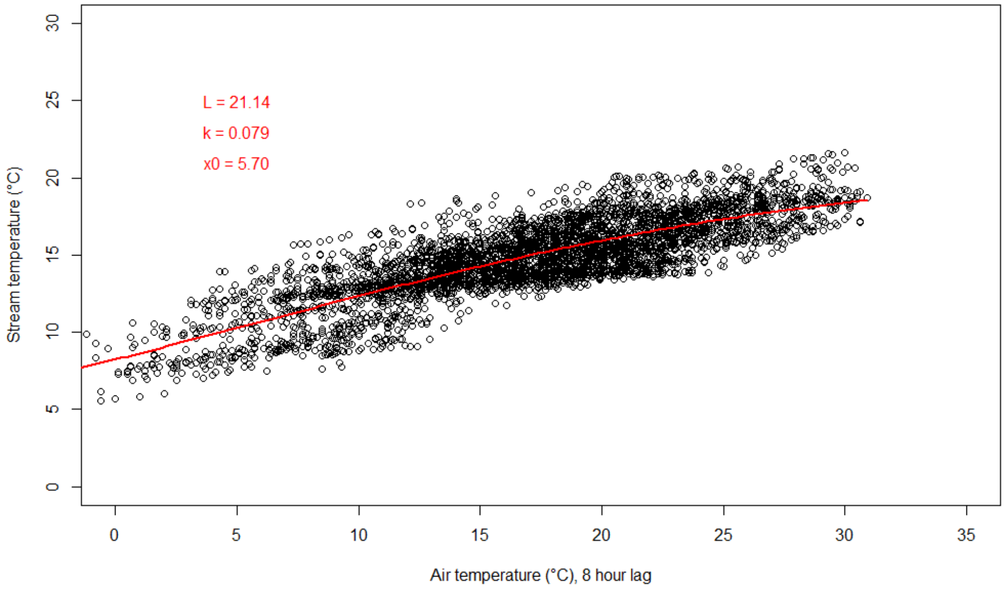

S4., The logistic relationship used to back-calculate stream temperature from air temperature data for Harper Creek. Temperature data used to build this relationship were from 2021. Variation in stream temperature was best explained by the air temperature eight hours prior.

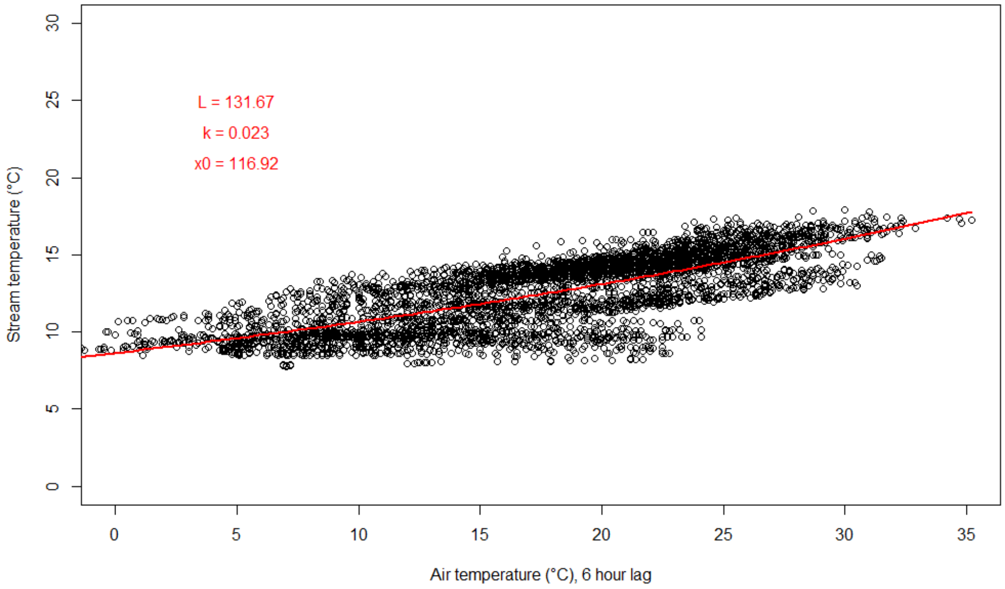

S4.n The logistic relationship used to back-calculate stream temperature from air temperature data for Oshawa Creek. Temperature data used to build this relationship were from 2012, 2013, and 2021. Variation in stream temperature was best explained by the air temperature six hours prior.

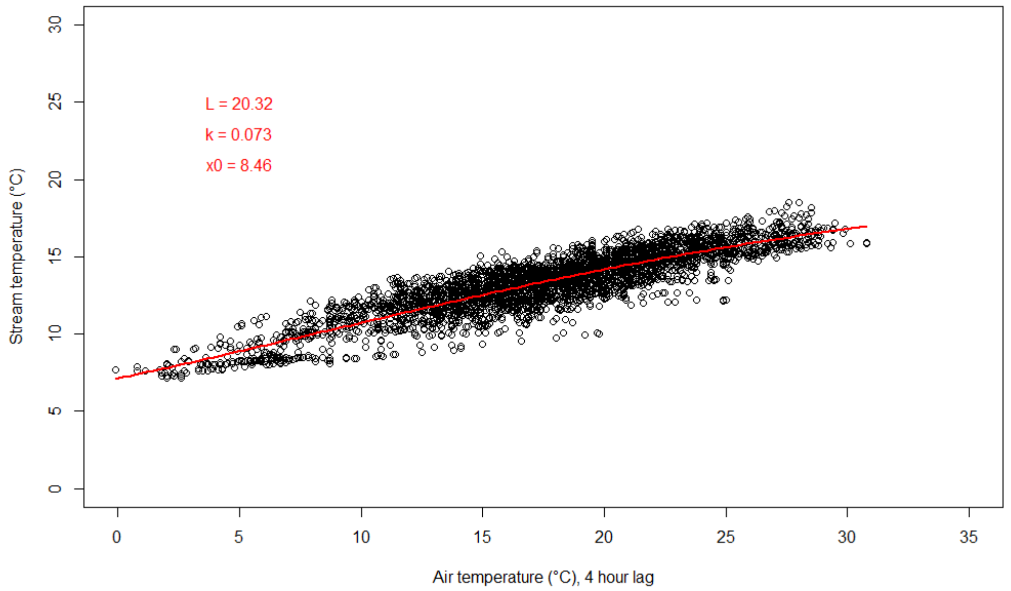

S4.o The logistic relationship used to back-calculate stream temperature from air temperature data for Pefferlaw Brook. Temperature data used to build this relationship were from 2021. Variation in stream temperature was best explained by the air temperature four hours prior.

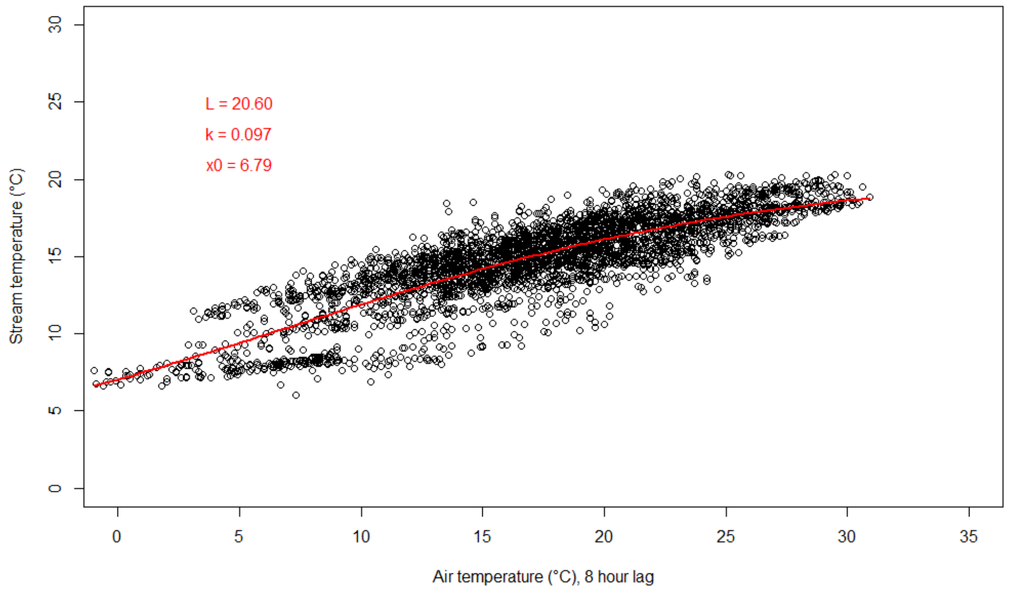

S4.p The logistic relationship used to back-calculate stream temperature from air temperature data for the Pigeon River (Main). Temperature data used to build this relationship were from 2021. Variation in stream temperature was best explained by the air temperature eight hours prior.

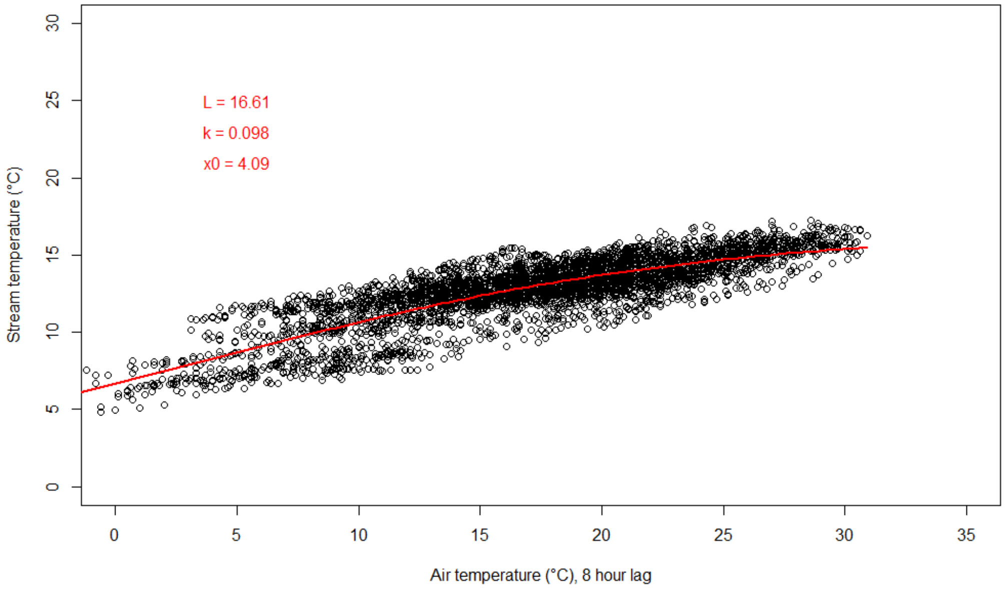

S4.q The logistic relationship used to back-calculate stream temperature from air temperature data for Pigeon River (Tributary). Temperature data used to build this relationship were from 2021. Variation in stream temperature was best explained by the air temperature eight hours prior.

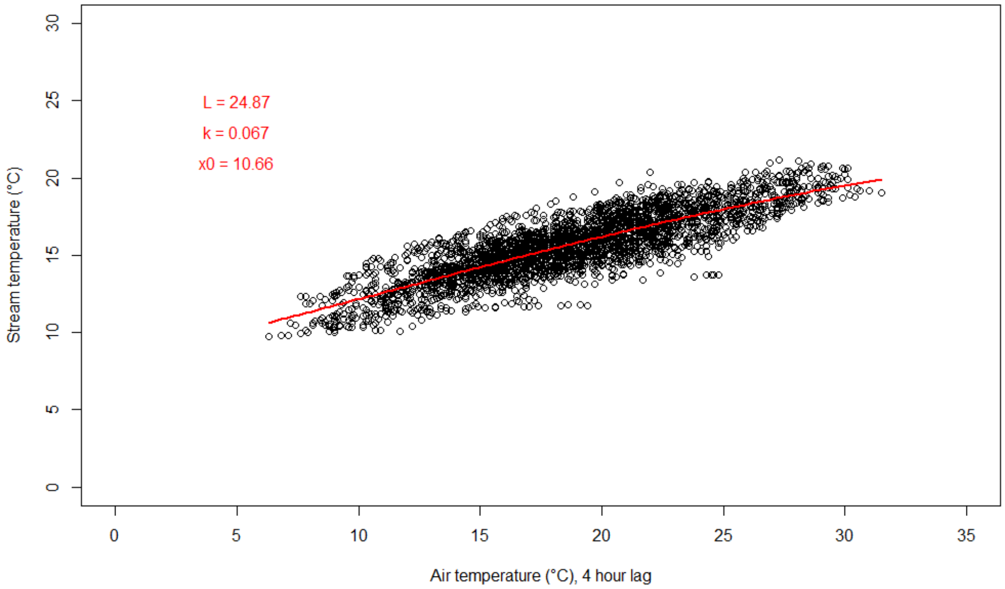

S4.r The logistic relationship used to back-calculate stream temperature from air temperature data for Pottageville Creek. Temperature data used to build this relationship were from 2021. Variation in stream temperature was best explained by the air temperature four hours prior.

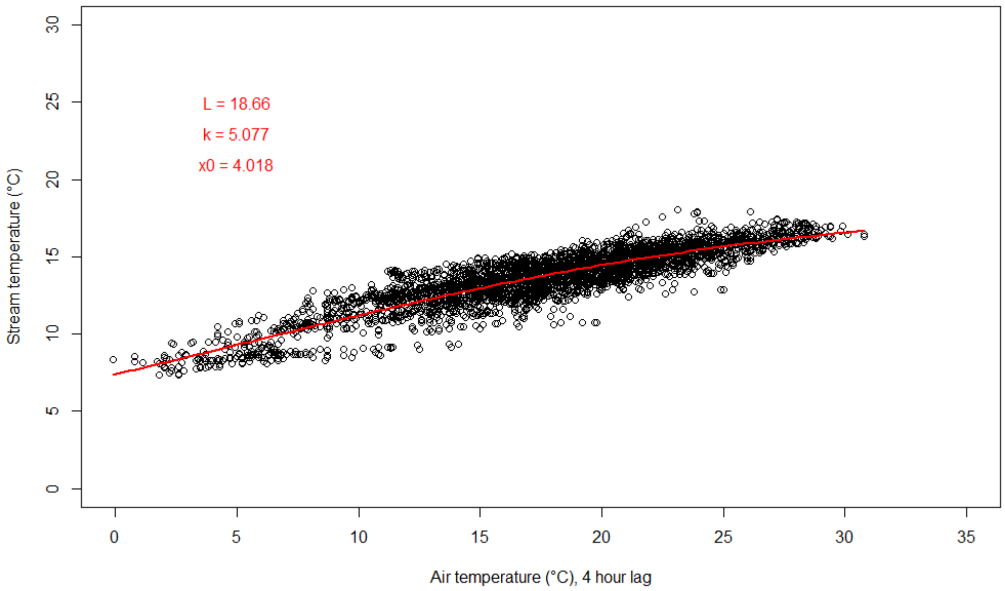

S4.s The logistic relationship used to back-calculate stream temperature from air temperature data for Uxbridge Brook. Temperature data used to build this relationship were from 2021. Variation in stream temperature was best explained by the air temperature four hours prior.

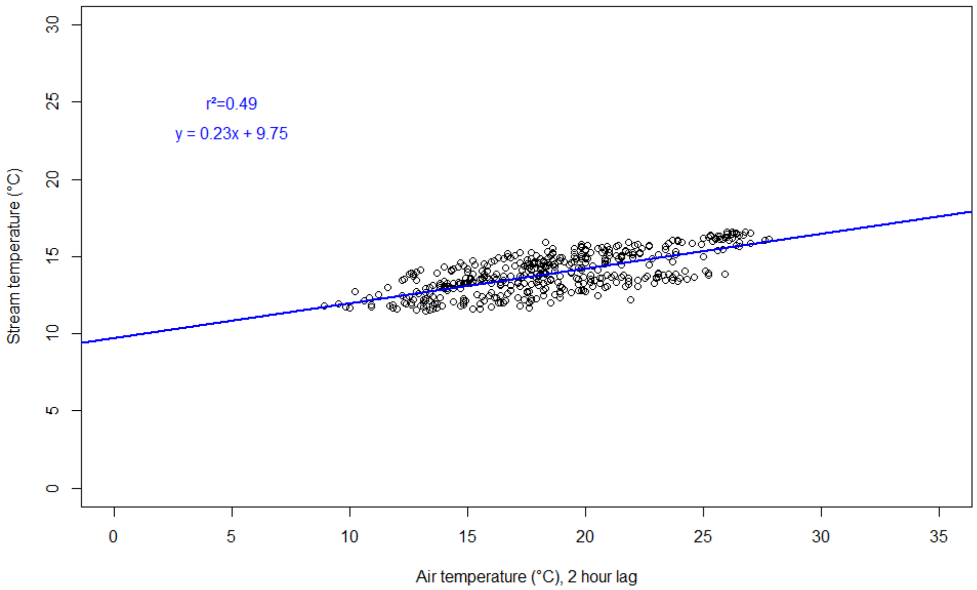

S4.t The linear relationship used to back-calculate stream temperature from air temperature data for the West Credit River. Temperature data used to build this relationship were from 2021. Variation in stream temperature was best explained by the air temperature two hours prior.

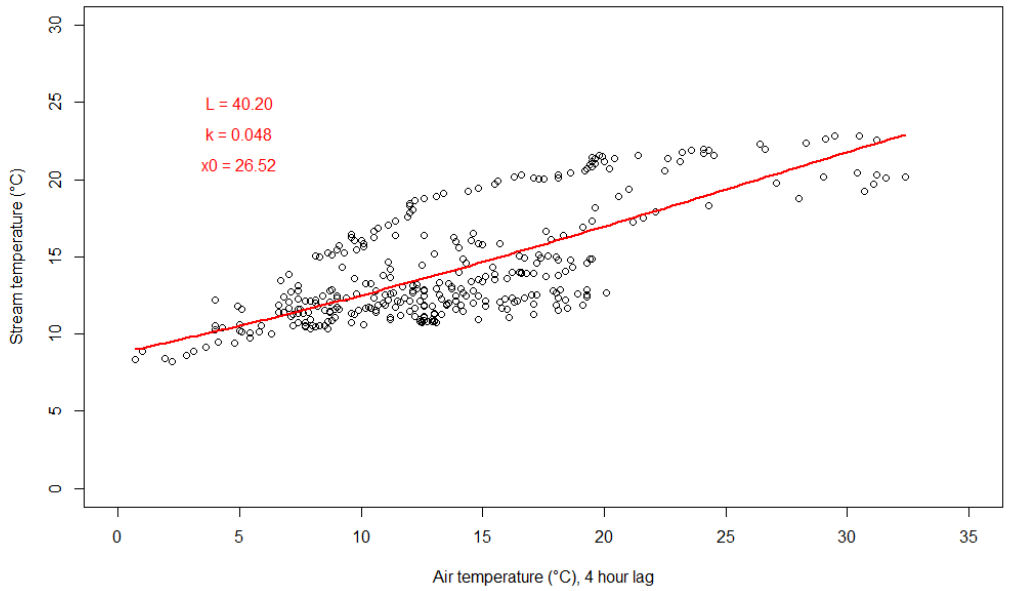

S4.u The logistic relationship used to back-calculate stream temperature from air temperature data for West Walkinshaw Creek. Temperature data used to build this relationship were from 2023. Variation in stream temperature was best explained by the air temperature four hours prior.

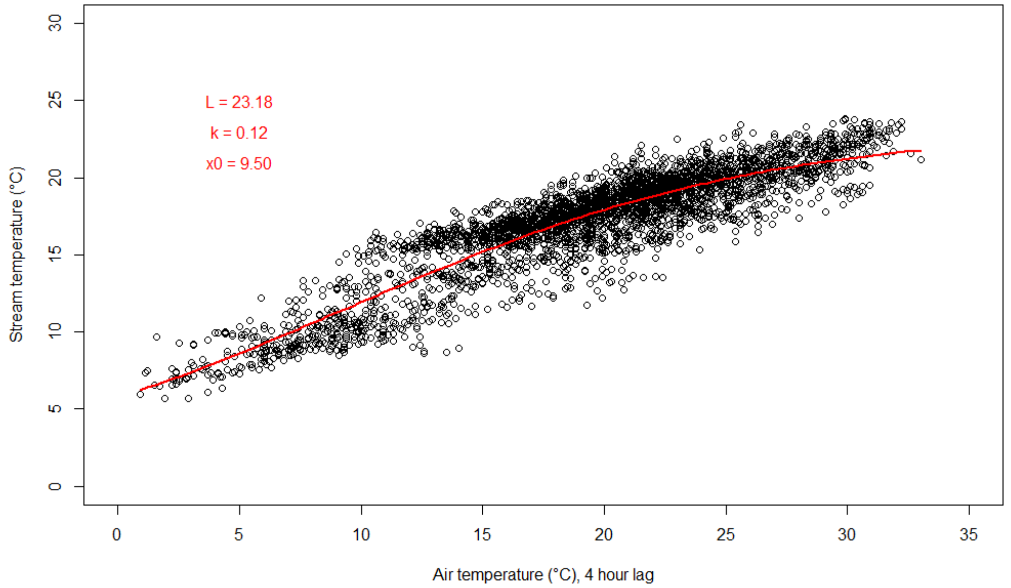

S4.v The logistic relationship used to back-calculate stream temperature from air temperature data for Willoughby Creek. Temperature data used to build this relationship were from 2021. Variation in stream temperature was best explained by the air temperature four hours prior.

S5 Overall effect of body size on CT_{max}. Slope and predicted values calculated from best-fit GAM (Model 1, Table 3). **A)** Predicted effect of body size (fork length, mm) on CT_{max} (GAM smooth; line) overlaid on raw data (points) with ticks marking the distribution of the raw data. **B)** Slope of the fitted function (i.e., first derivative of linear predictor, instantaneous rate of change) across the range of body sizes (fork length, mm) represented in the CT_{max} data at the mean 30-day acclimation temperature (14.63°C).

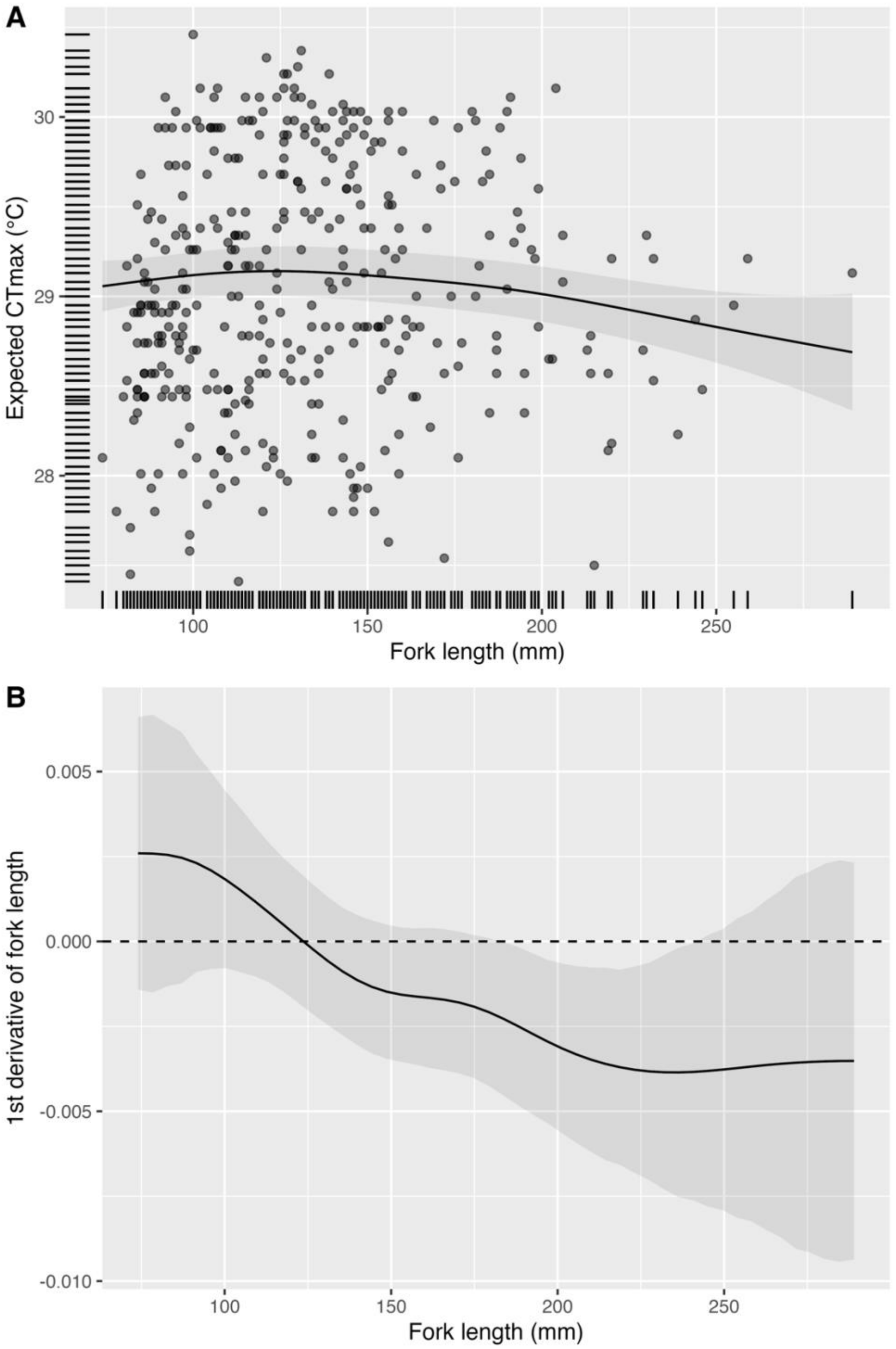

S6 Smoothed effect of sample day from candidate GAM to model seasonal effects of acclimation across dataset. Sample day (day of year) was modelled using cyclic cubic regression splines as a predictor of CT_{max} but demonstrated underfitting. Adjustment of basis dimensions (k) did not improve model fit. Site could not be included as a random effect in models with sample day (to account for site-level variation in seasonal thermal regimes to improve fit) as each site was sampled once and only one site was sampled per day.

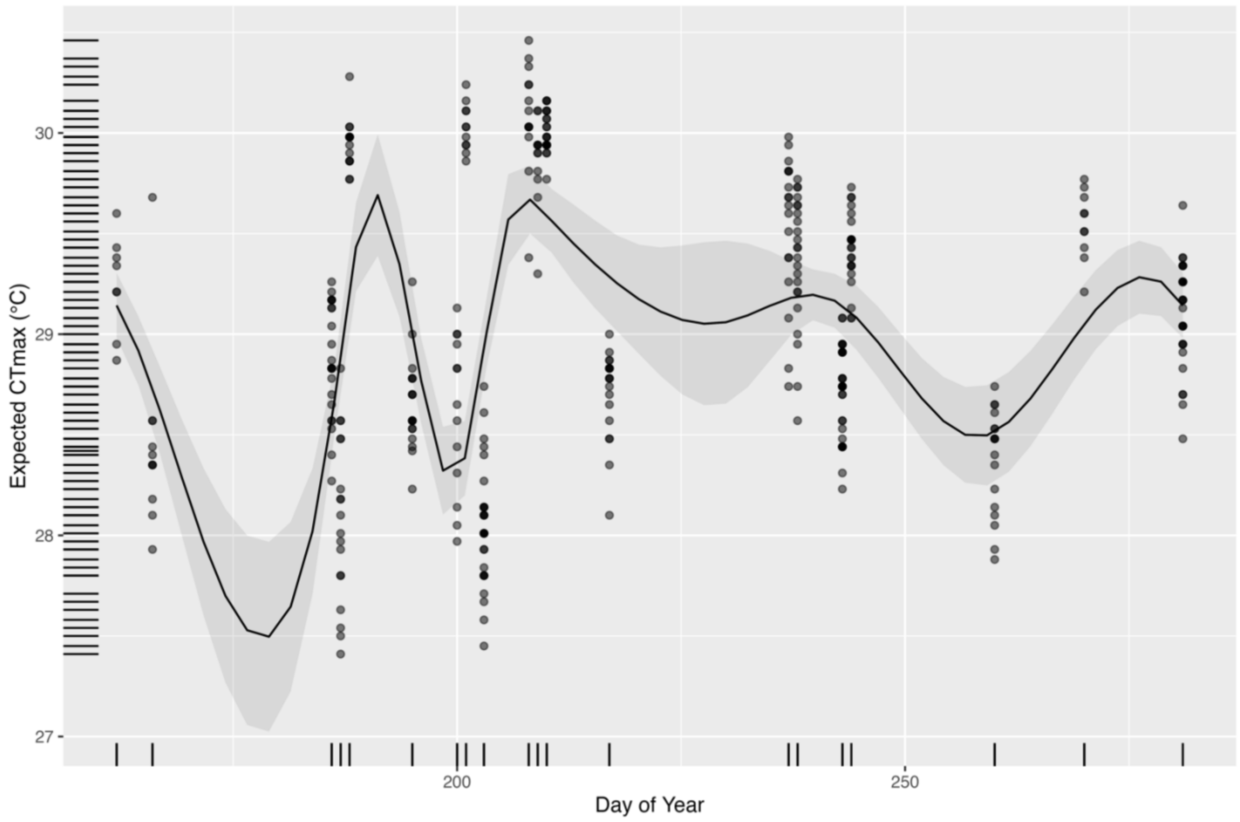

S7 Summary of brook trout field CT_{max} and acclimation data from 20 sites across Ontario, Canada. Sites are listed in alphabetical order. N indicates the number of fish tested. CT_{max} columns are in °C, length columns are in millimeters (mm), mass columns are in grams (g), and 'temp.' indicates temperature in °C. 'Overall' columns indicate values calculated using full season temperature data (May 1, 2021 – October 31, 2021). 30-Day columns indicate values calculated using the 30 day period prior to the trial date at a given site. Mean and max diel flux are the mean and maximum diel fluctuations at a site (maximum being within a given day). TSM is the calculated thermal safety margin (overall max temperature – site mean CT_{max}).

Site	Trial Date	N	CT_{max} mean	CT_{max} S.D.	CT_{max} S.E.	CT_{max} 95% CI	CT_{max} Range
Bowmanville	2021-09-01 11:45	24	29.43	0.18	0.04	0.07	0.65
Bronte	2021-07-19 11:30	13	28.61	0.39	0.11	0.24	1.16
Byersville	2021-08-26 11:15	23	29.36	0.32	0.07	0.14	1.2
Cavan	2021-10-08 12:00	33	29.11	0.25	0.04	0.09	1.16
Costello	2021-09-27 12:45	10	29.54	0.17	0.05	0.12	0.56
East Holland	2021-07-05 10:30	24	28.87	0.28	0.06	0.12	0.99
East Walkinshaw	2021-07-27 13:45	14	30.09	0.27	0.07	0.15	1.08
Fleetwood	2021-09-17 12:15	18	28.37	0.26	0.06	0.13	0.86
Furcate	2021-07-29 13:30	24	30.00	0.10	0.02	0.04	0.39
Ganaraska	2021-07-22 13:00	31	28.04	0.29	0.05	0.11	1.29
Harper	2021-08-25 12:15	18	29.54	0.36	0.09	0.18	1.24
Oshawa	2021-08-31 10:45	36	28.71	0.22	0.04	0.07	0.85
Pefferlaw	2021-07-06 10:45	18	28.07	0.41	0.10	0.20	1.42
Pigeon Main	2021-06-11 12:30	8	29.25	0.24	0.09	0.20	0.73
Pigeon Tributary	2021-06-15 12:00	11	28.45	0.45	0.14	0.30	1.75
Pottageville	2021-07-07 10:30	16	29.95	0.12	0.03	0.06	0.51
Uxbridge	2021-07-14 13:30	20	28.65	0.22	0.05	0.10	1.03
West Credit	2021-08-05 10:45	20	28.71	0.22	0.05	0.10	0.9
West Walkinshaw	2021-07-28 13:00	12	29.86	0.22	0.06	0.14	0.81
Willoughby	2021-07-20 13:00	11	30.03	0.12	0.04	0.08	0.38

Site	Length max	Length min	Length mean	Length S.D.	Mass max	Mass min	Mass mean	Mass S.D.	Length range	Mass range
Bowmanville	206	98	144.79	30.94	97.2	11.4	35.60	23.99	108	85.8
Bronte	172	86	119.85	26.76	51.4	6.9	21.56	14.49	86	44.5
Byersville	289	84	140.30	60.20	258.3	6.3	51.11	67.39	205	252
Cavan	230	81	124.24	44.21	153	5.3	29.25	36.96	149	147.7
Costello	259	91	156.90	48.22	194.4	7.5	52.49	53.40	168	186.9
East Holland	232	115	157.67	31.62	161.9	16.7	53.05	38.98	117	145.2
East Walkinshaw	180	95	124.93	24.19	69.6	10.4	25.31	16.55	85	59.2

Fleetwood	219	98	144.28	35.15	112.5	8.4	37.53	29.74	121	104.1
Furcate	156	90	116.58	18.61	48	8	19.93	10.45	66	40
Ganaraska	176	78	113.00	25.86	66.1	5.8	19.92	14.18	98	60.3
Harper	199	120	153.67	21.06	80.5	17.8	40.68	17.22	79	62.7
Oshawa	159	80	94.06	17.33	39.6	5.1	9.69	8.01	79	34.5
Pefferlaw	220	104	140.89	34.28	123.6	13.7	39.88	32.77	116	109.9
Pigeon Main	244	84	157.25	57.12	155.9	8.4	58.78	55.99	160	147.5
Pigeon Tributary	134	74	100.82	18.20	28.3	4.3	12.40	7.30	60	24
Pottageville	194	113	144.63	25.27	88.9	18.1	42.71	23.98	81	70.8
Uxbridge	246	89	153.60	51.05	146.7	9.7	54.10	44.61	157	137
West Credit	185	84	132.75	25.93	63.8	6.6	27.38	15.43	101	57.2
West Walkinshaw	176	102	124.83	21.54	56.1	11.1	21.93	12.60	74	45
Willoughby	204	114	149.82	26.69	95.6	16.4	43.17	25.40	90	79.2

**Site**	**Overall max temp.**	**Overall max date**	**Overall min temp.**	**Overall average temp.**	**Mean diel flux**	**Max diel flux**	**Mean daytime temp.**	**TSM**
Bowmanville	20.22	2021-06-05 19:00	6.38	14.15	3.89	8.53	14.61	9.21
Bronte	19.33	2021-06-30 16:00	5.57	10.81	1.76	8.31	11.20	9.28
Byersville	23.87	2021-08-13 22:00	6.26	15.72	2.77	7.59	15.42	5.49
Cavan	16.46	2021-08-23 20:00	11.14	13.94	1.92	3.42	14.03	12.65
Costello	25.39	2021-08-30 16:00	12.14	18.02	4.09	8.16	18.91	4.15
East Holland	20.59	2021-08-28 18:00	8.54	13.89	2.25	5.98	14.33	8.28
East Walkinshaw	24.16	2021-07-03 21:00	5.82	14.95	6.43	11.69	15.91	5.92
Fleetwood	14.25	2021-06-30 1:00	5.57	10.34	0.98	4.71	10.12	14.12
Furcate	29.49	2021-07-03 19:00	9.64	16.24	5.36	13.53	17.41	0.51
Ganaraska	12.53	2021-09-08 4:00	7.79	9.44	1.14	2.97	9.80	15.51
Harper	21.64	2021-06-28 22:00	5.55	14.62	2.91	6.78	14.37	7.90
Oshawa	16.56	2021-06-05 20:00	8.60	12.43	2.42	5.05	12.49	12.15
Pefferlaw	18.54	2021-06-28 16:00	6.53	12.97	2.35	6.61	13.54	9.52
Pigeon Main	20.31	2021-06-28 21:00	6.02	14.54	3.03	11.99	14.16	8.94
Pigeon Tributary	17.24	2021-06-27 20:00	4.78	12.53	2.18	6.05	12.24	11.21
Pottageville	21.16	2021-06-28 16:00	7.85	14.90	2.73	6.01	15.48	8.79
Uxbridge	18.07	2021-06-29 19:00	6.74	13.28	2.08	6.67	13.75	10.58
West Credit	16.59	2021-08-24 18:00	9.13	13.39	2.03	4.14	13.95	12.12
West Walkinshaw	23.78	2021-07-03 21:00	7.46	14.71	5.47	10.87	15.51	6.09
Willoughby	23.85	2021-06-28 16:00	5.65	16.56	4.02	10.32	17.53	6.18

Site	30-Day Temp. Mean	30-Day Temp. S.D.	Seasonal Temp. Range	30-Day Temp. Max	30-Day Temp. Max Date
Bowmanville	16.36	2.57	13.84	19.61	2021-08-09 20:00
Bronte	12.37	1.70	13.76	19.33	2021-06-30 16:00
Byersville	17.59	2.99	17.61	23.87	2021-08-13 22:00
Cavan	13.64	1.09	5.32	15.45	2021-09-17 21:00
Costello	17.51	2.65	13.25	25.39	2021-08-30 16:00
East Holland	14.64	1.98	12.05	19.81	2021-06-27 17:00
East Walkinshaw	17.40	3.74	18.35	24.16	2021-07-03 21:00
Fleetwood	11.56	1.58	8.69	14.03	2021-08-30 6:00
Furcate	18.63	3.24	19.86	29.49	2021-07-03 19:00
Ganaraska	9.09	0.77	4.75	10.69	2021-06-28 16:00
Harper	16.25	2.70	16.10	20.08	2021-08-14 1:00
Oshawa	12.97	1.43	7.96	16.44	2021-08-30 3:00
Pefferlaw	14.60	2.26	12.01	18.54	2021-06-28 16:00
Pigeon Main	14.73	2.96	14.29	19.87	2021-06-09 20:00
Pigeon Tributary	12.35	2.26	12.46	16.88	2021-06-09 21:00
Pottageville	16.27	2.56	13.31	21.16	2021-06-28 16:00
Uxbridge	14.34	2.13	11.33	18.07	2021-06-29 19:00
West Credit	13.91	1.45	7.46	16.58	2021-07-25 15:00
West Walkinshaw	16.69	3.11	16.31	23.78	2021-07-03 21:00
Willoughby	18.42	3.54	18.19	23.85	2021-06-28 16:00
